# Waterlogging affects plant morphology and the expression of key genes in tef (*Eragrostis tef*)

**DOI:** 10.1002/pld3.56

**Published:** 2018-04-25

**Authors:** Gina Cannarozzi, Annett Weichert, Mirjam Schnell, Celia Ruiz, Svenja Bossard, Regula Blösch, Sonia Plaza‐Wüthrich, Solomon Chanyalew, Kebebew Assefa, Zerihun Tadele

**Affiliations:** ^1^ Institute of Plant Sciences University of Bern Bern Switzerland; ^2^ Swiss Institute of Bioinformatics Lausanne Switzerland; ^3^ Kantonsschule Zürich Nord Zürich Switzerland; ^4^ Ethiopian Agricultural Research Institute Debre Zeit Agricultural Research Center Debre Zeit Ethiopia; ^5^ Institute of Biotechnology Addis Ababa University Addis Ababa Ethiopia; ^6^ Present address: Département des Neurosciences Cliniques Centre Hospitalier Universitaire Vaudois Lausanne Switzerland

**Keywords:** adventitious roots, aerenchyma, differential expression, *Eragrostis tef*, flooding, grass family, RNA‐Seq, tef, transcriptome, waterlogging

## Abstract

Tef [*Eragrostis tef* (Zucc.) Trotter], an allotetraploid cereal that is a staple food to over 60 million people in the Horn of Africa, has a high nutritional content and is resistant to many biotic and abiotic stresses such as waterlogging and drought. Three tef genotypes, *Alba*,* Tsedey*, and *Quncho*, were subjected to waterlogging conditions and their growth, physiology, and change in transcript expression were measured with the goal of identifying targets for breeding cultivars with improved waterlogging tolerance. Root and shoot growth and dry weight were observed over 22 days. Stomatal conductance and chlorophyll and carotenoid contents were quantified. Microscopy was used to monitor changes in the stem cross sections. Illumina RNA sequencing was used to obtain the expression profiles of tef under flooding and control conditions and was verified using qPCR. Results indicated differences in growth between the three genotypes. Waterlogged *Tsedey* plants grew higher and had more root biomass than normally watered *Tsedey* plants. *Quncho* and *Alba* genotypes were more susceptible to the excess moisture stress. The effects of these changes were observed on the plant physiology. Among the three tested tef genotypes, *Tsedey* formed more aerenchyma than *Alba* and had accelerated growth under waterlogging. *Tsedey* and *Quncho* had constitutive aerenchyma. Genes affecting carbohydrate metabolism, cell growth, response to reactive oxygen species, transport, signaling, and stress responses were found to change under excess moisture stress. In general, these results show the presence of substantial anatomical and physiological differences among tef genotypes when waterlogged during the early growth stage.

## 1 INTRODUCTION

Climate change is one of the most challenging problems facing global food security. Along with changes in temperature, the frequency and severity of both extreme droughts and extreme precipitation events are expected to increase (Hartmann, Tank, & Rusticucci, 2013) causing billions of dollars of crop losses (Bailey‐Serres, Lee, & Brinton, 2012). Understanding of the genetic basis of the plant response to abiotic constraints is critical for the development of cultivars that are resilient to stress and to decrease the gap between yield potential and actual yield under stressed conditions (Setter & Waters, 2003).

Tef [*Eragrostis tef* (Zucc.) Trotter], an allotetraploid cereal crop, is the major crop in the Horn of Africa, especially in Ethiopia where it is annually cultivated on over three million hectares of land and is staple food to over 60 million people (CSA 2015). Of the approximately 350 members of the *Eragrostis* genus, tef is the only one cultivated for use as a human food although several species are used as livestock fodder or forage grasses (Ketema, 1993). Tef grows in wide agroecological conditions ranging from semiarid areas with low rainfall to areas with high rainfall. It is also a dominant crop in poorly drained soils commonly known as *vertisols* which cover about 10% of the total area of Ethiopia, much of which are in high‐rainfall areas (Mekonen, Tesfaye, & Bayu, 2013). The combination of poorly drained soil and high rainfall is conducive to waterlogging and is responsible for both low yields and underutilization of this land for farming (Asamenew, Beyene, Negatu, & Ayele, 1993).

Waterlogging is a soil condition in which excess water causes inadequate gas exchange between the soil and the atmosphere. The effects on the plant are manifold. Oxygen diffuses approximately 10,000 times slower in water than in air, and the flow of oxygen into waterlogged soil is around 320,000 times less (Watanabe, Nishiuchi, Kulichikhin, & Nakazono, 2013). Therefore, the amount of oxygen available to the roots decreases (Jackson & Colmer, 2005; Lee et al., 2011), and photosynthesis and respiration are limited. Waterlogging can decrease a cell's resistance to pathogens (Hsu et al., 2013). Additionally, phytotoxic compounds such as sulfides and the reduced forms of minerals (e.g., Mn^2+^ and Fe^2+^) accumulate in waterlogged soil (Laanbroek, 1990; Nishiuchi, Yamauchi, Takahashi, Kotula, & Nakazono, 2012), creating oxidative stress (Fukao, Yeung, & Bailey‐Serres, 2011).

Waterlogging‐tolerant plants have many morphological responses to these stresses. A key response of many plants to flooding is the formation of adventitious roots, roots that form from non‐root tissue (Steffens & Rasmussen, 2016). In rice, excess water triggers a signal cascade involving many hormones including ethylene, abscisic acid (ABA), gibberellic acid (GA), auxin (Pacurar, Perrone, & Bellini, 2014), and cytokinin (Dat, Capelli, Folzer, Bourgeade, & Badot, 2004). Ethylene is unable to escape, and the consequent buildup of ethylene triggers adventitious root growth (Lorbiecke & Sauter, 1999). The newly formed adventitious roots often contain aerenchyma, air‐filled spaces that provide an internal pathway for the movement of oxygen from the well‐aerated shoots to the roots. Wetland plant species often possess constitutive aerenchyma or can form aerenchyma upon flooding (Colmer & Pedersen, 2008; Yamauchi, Shimamura, Nakazono, & Mochizuki, 2013), while in many dryland flooding‐tolerant species, waterlogging induces aerenchyma formation (Colmer & Voesenek, 2009). Several studies have correlated the formation of aerenchyma with waterlogging tolerance (Abiko et al., 2012; Herzog, Striker, Colmer, & Pedersen, 2016; Setter & Waters, 2003; Zhang et al., 2015).

Another morphological adaptation to flooding is the formation of barriers to radial oxygen loss (ROL) in the basal part of the root to minimize oxygen loss to the environment and keep the oxygen moving toward the root apex (Abiko et al., 2012; Colmer, Cox, & Voesenek, 2006; Nishiuchi et al., 2012; Watanabe et al., 2013). These barriers may also impede the movement of soil toxins and gases into the root (Armstrong, 1979; Colmer, 2003; Watanabe et al., 2013). Both suberin (Watanabe et al., 2013) and lignin (Kotula, Ranathunge, Schreiber, & Steudle, 2009) may be components of this apoplastic barrier. A hybrid between wheat (*Triticum aestevum*) and a wild waterlogging‐tolerant barley with constitutive aerenchyma and an inducible ROL barrier was found to be more waterlogging tolerant than the parental wheat line (Malik, Islam, & Colmer, 2011). Similarly, aerenchyma formation combined with an inducible barrier to ROL contributed to the waterlogging tolerance of a newly discovered teosinte (*Zea nicaraguensis*) compared to cultivated maize (*Zea mays*) (Abiko et al., 2012).

The production of reactive oxygen species (ROS) is a consequence of aerobic metabolism and may be a part of the signaling that stimulates the growth of adventitious roots (Steffens & Rasmussen, 2016). Detoxification of ROS is mediated by both enzymatic (superoxide dismutase, ascorbate peroxidase, etc.) and non‐enzymatic (carotenoids, ascorbic acid, glutathione) antioxidants (Lekshmy, Jha, & Sairam, 2015). In crop plants, upregulation of antioxidants has been found to be correlated with abiotic stress tolerance (Rivera‐Contreras et al., 2016; Ushimaru et al., 1997; Zhang et al., 2015) and has even been suggested to be the basis for waterlogging tolerance (Thirunavukkarasu et al., 2013).

Two strategies employed by rice to combat flood are (i) *escape*, rapid growth of the shoots that allows the plant to reach the surface as quickly as possible, and (ii) *quiescence*, entering a state of inactivity until the flooding conditions have passed (Nishiuchi et al., 2012). Both strategies are mediated by ethylene. *Escape* is a useful response in the case of deep and long‐lasting flooding, while *quiescence* is employed for shallower floods of short duration. Submergence‐tolerant rice cultivars employing the *quiescence* strategy restrict the consumption of carbohydrates, retain chlorophyll to maintain limited photosynthesis, and express an ethylene‐responsive factor (ERF) gene, Submergence 1A (SUB1A), which confers submergence tolerance by dampening ethylene production and GA responsiveness (Fukao, Xu, Ronald, & Bailey‐Serres, 2006; Xu et al., 2006). In deepwater rice, the *escape* strategy is employed. When ethylene accumulates in tissues, it stimulates internode elongation (Hattori et al., 2009; Kende, van der Knaap, & Cho, 1998; Raskin & Kende, 1983), via two ethylene‐responsive factors, Snorkel1 (SK1) and Snorkel2 (SK2) (Fukao et al., 2006). To allow rapid shoot elongation, proteins such as expansins are expressed to loosen the cell wall (Li, Jones, & McQueen‐Mason, 2003). Rapid shoot elongation under hypoxic conditions (*escape* strategy) has also been reported for several other plants including arrowhead (*Sagittaria pygmaea* Miq.), pondweed (*Potamogeton distinctus* A. Benn) (Ishizawa, Murakami, Kawakami, & Kuramochi, 1999; Summers & Jackson, 1994), and two rumex species (*Rumex Palustris* and *Rumex acetosa*) (van Veen et al., 2013) although it has been suggested that rapid shoot growth is detrimental to plant growth after the floodwaters recede (Kawano, Ito, & Sakagami, 2009).

The physiological response of plants subjected to waterlogging stress involves a switch from aerobic respiration to fermentative metabolism, and consequently, genes that are involved in starch breakdown such as the glycolytic and fermentative pathways are affected (Parent, Capelli, Berger, Crèvecoeur, & Dat, 2008). Tolerant plants can also photosynthesize and respire while flooded (Caudle & Maricle, 2012). Thus, physiological measurements such as stomatal conductance and leaf chlorophyll content are valuable probes of the plant response.

The Tef Improvement Project was established to increase tef productivity using modern plant breeding methods and has undertaken genome and transcriptome sequencing for this purpose (Cannarozzi et al., 2018). Here, we report a study on the response to waterlogging of three tef genotypes—a landrace, *Alba*, and two improved varieties *Tsedey* and *Quncho*. *Quncho*, a cross between *Dukem* (maternal) and *Magna* (paternal), is a popular variety as it combines the high yield of *Dukem* with the high seed quality of *Magna*. *Tsedey*, an improved variety on the market since 1984, can grow in a wide range of soil, water, and air conditions. Here, growth and biomass have been measured for tef subjected to early (at 4 days) waterlogging. In addition, we combined an expression study using RNA‐Seq and qPCR with physiological studies and microscopy to understand the response of tef to waterlogging at the tillering stage. The results show that there are substantial differences in growth, waterlogging tolerance, anatomy, and physiology between the three genotypes studied in the early and tillering stages of waterlogging, diversity that is necessary for breeding the trait into new germplasm and identification of the responsible genes using association mapping. The most tolerant genotype, *Tsedey*, has greater growth of both the shoot and the root, more constitutive aerenchyma, and aerenchyma production. Genes affecting carbohydrate metabolism, cell growth, response to reactive oxygen species, transport, signaling, and stress responses were found to change under stress in the *Tsedey* improved variety, consistent with the physiological and anatomical changes observed.

## 2 MATERIALS AND METHODS

Experiments designed to monitor root and shoot growth, aerenchyma size, physiology, and gene expression are outlined in Table 1.

**Table 1:** Summary of growth, expression, and physiology experiments conducted

	Experiment type	Genotypes	Number of plants of each genotype	Tissue	Age of plant during treatment	Evaluation methods	Parameters measured	Stage Figure or Table
1	Growth 1	*Alba, Quncho, Tsedey*	18	Roots, shoots	From 4 to 28 days	Measure shoot length in pots	Shoot length	Early stage Figure S1
2	Growth 2	*Quncho, Tsedey*	66	Roots, shoots	From 4 to 28 days	Harvest 4 controls and 4 waterlogged at 2, 4, 7, 9, 11, 16, and 24 days after treatment	Root and shoot length, dry weight, cross section	Early stage Figures 1 and S6 Tables 2, S1, S2
3	Root growth 1	*Alba, Quncho, Tsedey*	2	Roots	From 4 to 22 days	Visualization with rhizotron	Root growth	Early stage Figure S2
4	Root growth 2	Wheat, maize, *Alba, Tsedey*	2	Roots, shoots	From 7 to 35 days	Visualization with rhizotron	Root growth	Early stage Figures S3 (wheat) and S4 (maize)
5	Physiology	*Tsedey*		Shoots	From 19 to 28 days	Porometer SPAD	Chlorophyll a and b, carotenoid, stomatal conductance	Tillering stage Figure 2
6	Cross sections	*Alba, Quncho, Tsedey*	21	Roots	From 19 to 28 days	Count adventitious roots, cross sections	Number of adventitious roots Aerenchyma size	Tillering stage Figures 3, 4 and S5 Table S3
7	qPCR	*Alba, Quncho, Tsedey*		Shoots	From 19 to 28 days	Microscopy, qPCR	Differential gene expression	Tillering stage Figure 5 Table S6
8	RNA‐Seq	*Tsedey*		Shoots	From 19 to 28 days	RNA‐Seq	Differential gene expression	Tillering stage Figures S7, S8, S9, S10, S11, and S12 Tables 3, 4, 5, S4, and S5

### 2.1 Tef genotypes used

Three *Eragrostis tef* genotypes used in these experiments were as follows: (i) *Tsedey* (DZ‐Cr‐37), an improved variety whose genome and transcriptome have been sequenced and which is thought to be waterlogging resistant, (ii) *Alba*, a landrace obtained from the US Department of Agriculture (USDA), and (iii) *Quncho* (DZ‐Cr‐387 RIL‐355), a popular high‐yielding and white‐seeded variety. *Tsedey* and *Quncho* were obtained from the Ethiopian Institute of Agricultural Research (EIAR).

### 2.2 Growing conditions for Experiment 1 (Growth 1): plant height

Three seeds of tef genotypes (*Alba*,* Tsedey*, and *Quncho*) were sown in pots and grown under long‐day conditions (16‐h light at 22°C and 65% relative humidity; 8‐h dark at 18°C and 65% relative humidity). For the waterlogging treatment, the pots were put in a plastic tray in which the water level was maintained at 1 cm below the surface of the soil. The control plants were put in an identical tray and watered from below every 3 days followed by drainage of the tray. The waterlogging treatment was applied to 18 (9 controls, 9 waterlogged) 4‐day‐old plants. Day 0 marked the first day of treatment. Plant height (from the base of the root to the end of the longest leaf) was recorded for the next 24 days, namely at 0, 1, 3, 4, 7, 8, 14, 17, and 22 days after waterlogging.

### 2.3 Growing conditions for Experiment 2 (Growth 2): Early waterlogging time series of plant height, root length, and root/shoot dry weight

Experiment 2 is a repetition of Experiment 1 using 66 seedlings of *Tsedey* and *Quncho* sown in pots and grown under long‐day conditions as indicated above. For the waterlogging treatment, the pots were put in a plastic tray in which the water level was maintained at 1 cm below the surface of the soil. The control plants were put in an identical tray and watered from below every 3 days followed by drainage of the tray. Four days after germination, treatment started and the plants were grown for the next 24 days under control or waterlogging conditions. At 2, 4, 7, 9, 11, 16, and 24 days after treatment, four plants were harvested, one root was reserved for cross‐section, the remaining roots washed under tap water, the separated roots and shoots were dried at 60°C overnight, and the root and shoot dry weight and length were measured.

### 2.4 Growing conditions for Experiment 3: tef root visualization using rhizotrons

In experiment 3, seeds of *Alba*,* Quncho*, and *Tsedey* were grown in Plexiglass rhizotrons with dimensions of 20 cm wide, 30 cm high, and 1.5 cm deep. After germination, all plantlets except the one with the most visible root were culled. Water was available from the bottom of the rhizotron or could leak in at the seals on the sides. For the waterlogging treatment, the water level was maintained at 1 cm below the soil surface. The control plants were watered from the bottom and moistened at the top. When not being photographed, the rhizotrons were kept in a box with a lid that protected them from the light.

### 2.5 Growing conditions for Experiment 4: wheat and maize root visualization using rhizotrons

In experiment 4, bobwhite wheat and Akku maize varieties were obtained from the group of Matthias Erb at the Institute of Plant Sciences at the University of Bern. The seeds of wheat and maize were soaked in 2.5% bleach solution for 3–4 min and then repeatedly rinsed while agitating. They were then planted on wet paper towels in Petri dishes and then transferred to the rhizotrons after 4 days. Treatment was started one week after transplantation into the rhizotron. The waterlogging conditions were the same as for Experiment 3.

### 2.6 Growing conditions for Experiment 5 (Physiology) and Experiment 8 (RNA‐Seq)

Seedlings of the improved tef variety *Tsedey* were grown under long‐day conditions (16 h of light at 24°C and 8 h of dark at 18°C, 65% relative humidity) in pots. The seedlings were grown for 19 days and then exposed for 9 days to two conditions (waterlogging and normal watering). Waterlogging was achieved by maintaining the water level at 1 cm below the soil surface. After 9 days of treatment, the stomatal conductance, chlorophyll a and b, and carotenoid content were measured, the plants were harvested, and RNA was extracted from leaf tissue (described below).

### 2.7 Growing conditions for cross sections at the tillering stage Experiment 6 and Experiment 7 (qPCR)

*Alba, Tsedey* and *Quncho* varieties were sown in 7.5‐cm‐diameter pots and grown under long‐day conditions in the growth room as indicated above. Waterlogging treatment (water level maintained at 1 cm below the soil surface) was applied for 9 days to 19‐day‐old plants. After 9 days of growth under control or treatment conditions, the plants were harvested, the wet weight of the root and shoot was measured, the number of adventitious roots per plant was counted, plant material was collected for qPCR, and one or two adventitious roots were taken for cross sections. This procedure was performed in duplicate, both times with 10 or 11 plants (observation 1 and observation 2). For the cross sections, sometimes more than one cross section from the same plant and root location were taken which were considered as technical replicates and hence were not counted as independent observations. At the middle of the root, it was often difficult to get an unblemished cross section. The filenames for the cross sections are named for the plant (T: for *Tsedey*, Q: for *Quncho*, or A: for *Alba*), the number of the plants (1‐17), the location on the root (oben (root base), mitte (middle), or unten (tip)), the replicate (1‐5), and the magnification.

### 2.8 Physiological measurements

Stomatal conductance of the flag leaf for the adaxial side was determined using an AP4 diffusion porometer (Delta T, Cambridge Life Sciences, Cambridge, UK) at the end of the water stress period. Chlorophyll a and b, as well as carotenoids which comprise carotenes and xanthophylls, were extracted using 95% ethanol and measured with UV–Vis spectroscopy as previously described (Lichtenthaler, 1987). The amount of these pigments was normalized by fresh weight. For the physiological measurements, 10 measurements from 10 different plants were used.

### 2.9 Statistical analysis for the physiology and growth measurements

Data are presented as means ± standard deviation. Statistical tests were made using R version 3.0.1 using the built‐in function wilcox.test. First, an ANOVA test was used to determine whether significant differences existed. If so, outliers were removed using a modified Thompson tau test. Nonparametric tests (Mann–Whitney *U*‐test or Kruskal–Wallis H tests) with a *p*‐value of ≤.05 were used to determine statistical significance between treatment means.

### 2.10 RNA extraction for RNA‐Seq

RNA was extracted from samples of leaf tissue using the TRIzol kit (ThermoFisher Scientific) according to the supplier's protocol. The quality and quantity of RNA were quantified using ND‐1000 spectrophotometer for which the average 260/280 ratio was 2.0, indicating good‐quality RNA.

### 2.11 Transcriptome library construction and sequencing for RNA‐Seq

The RNA extracted from plants grown under waterlogging and normal watering conditions was sent to Fasteris (Geneva, Switzerland, www.fasteris.com) for further quality testing and sequencing using Illumina HiSeq2000 with the goal of analysis for differential expression.

Two biologic replicates were made leading to two libraries for control (GNY1 and GNY10) and two from the waterlogged plants (GNY3 and GNY12). The GNY10 and GNY12 libraries were prepared using the AccuPrime polymerase (Invitrogen, Carlsbad, CA) and following the protocol for high GC content. The GNY1a and GNY3a samples were prepared with the TruSeq SBS version 5 kit and the data analysis pipeline consisting of HiSeq Control Software version 1.1.37.8, RTA 1.7.48, and CASAVA 1.7. GNY1b, GNY3b, GNY10, and GNY12 also used the TruSeq SBS version 5 kit and flow cell version 3 with software: HiSeq Control Software version 1.4.8, RTA 1.12.4.2, and CASAVA 1.8.2. The six cDNA libraries were sequenced to generate a total of 205 million single‐end reads. Before assembly, the reads were trimmed such that the Phred quality scores were above 28. In addition, all primer and adaptor sequences detected by FastQC were removed.

### 2.12 Quantification of gene expression levels and differential expression experiments

The reads from each condition were mapped onto the 14,057 scaffolds of size 1000 bp or greater obtained from the recently sequenced tef genome (Cannarozzi et al., 2014) using STAR 2.3.0 with the default parameters. These were converted to BAM format with SAMtools (Li et al., 2009).

A count table was obtained using the HTSeq‐count program with options stranded=no, type=gene, and attribute=ID (Anders, Pyl, & Huber, 2015) and using the Maker gene predictions provided by the Tef Genome Project (Et_genome_1.0.fasta.gz) (Cannarozzi et al., 2014). HTSeq tabulates the percentage of uniquely mapping reads, reads that map to no feature in the predicted transcriptome, ambiguous reads (map to more than one gene simultaneously), and reads that do not map uniquely to one location. Only uniquely mapping reads were used for counting. The newCountDataSetFromHTSeqCount function of the DESeq package was used to generate the count table used as input into DESeq, a Bioconductor package that estimates the variance–mean dependence of high‐throughput sequence data and tests for differential expression using the negative binomial model (Anders & Huber, 2010).

### 2.13 Annotation and enrichment analysis

#### 2.13.1 Background set

The transcriptome used in these experiments was from the file Et_all.maker.transcripts.shortids.fasta.gz provided by the Tef Improvement Project. This file contains genes predicted in the genome by Maker and described in (Cannarozzi et al., 2014). To define a background set for the enrichment analysis, the reads were mapped onto the set of 42,052 genes predicted from the tef genomic scaffolds, creating a background set consisting of all 34,761 genes detected in the experiment. The background set was annotated using Blast2GO with the default parameters (E‐value 1.0 e‐03, blastp, non‐redundant database at NCBI). Differentially regulated genes were defined as those found by DESeq to have an adjusted *p*‐value of less than 0.05 and a fold change of 2 or more. Assignment and clustering of GO terms produced biologic process, molecular function, and cellular component of these differentially regulated terms. Enrichment analysis using Fisher's exact test at α = 0.05 was used to determine genes differentially expressed.

#### 2.13.2 Mercator

Sequences were also placed into MapMan functional categories based on the sequence similarity using the Web interface to Mercator (http://mapman.gabipd.org/web/guest/mercator) (Lohse et al., 2014) using annotated sequences from the following: the Arabidopsis Information Resource (TAIR) (Berardini et al., 2015), SwissProt/UniProt plant proteins (Bateman et al., 2017), TIGR5 rice proteins (Kawahara et al., 2013) and the COG database (Tatusov et al., 2003) and BLAST cutoff 80. Multiple bin assignments were allowed. Enrichment analyses of various pathways using a Wilcoxon rank‐sum test with Benjamini–Hochberg corrections for multiple testing were conducted using a local installation of the MapMan software.

### 2.14 Verification of expression levels using qPCR

Plant material was collected as described above and was then ground in liquid nitrogen. Total RNA was extracted using the Total RNA Isolation System (Promega, USA) and treated with the DNA‐free kit from Life Technologies, USA. RNA was quantified using a NanoDrop ND‐1000 spectrophotometer (Thermo Scientific, USA). For calculating the relative gene expression, the 2‐ΔΔCq method was used (Livak & Schmittgen, 2001). CYP and PP2A were used as reference genes. Each biologic replicate was sampled three times, and the measured Cq values were averaged.

### 2.15 Preparation and microscopy of cross sections of adventitious roots

The adventitious roots were cut into three sections of equal length, referred as “base” (closest to the shoot), “middle,” and “tip” (closest to the root tip or apex). These were embedded in 5% agarose and sliced into 100‐ to 200‐μm slices using a Vibratome. No staining or dyes were used. The cross sections were visualized in a Zeiss Axioskop 2 microscope with an AxioCam color (412–312) camera using 5× to 20× magnification. The microscope software was AxioVision Release 4.8.2 SP2 (06‐2012), and ImageJ (Duarte et al., 2016) was also used in the image processing.

### 2.16 Quantification of aerenchyma

Processing of the cross sections with Photoshop included manually selecting the cortex and aerenchyma.

$$\%\;\text{Aerenchyma}=\left(1-\frac{\text{Cortex area}-\text{Aerenchyma area}}{\text{Cortex area}}\right)\times 100$$

### 2.17 Phylogeny

Sequences were aligned using Mafft (L‐INS‐I) (Katoh & Standley, 2013) with the default settings. PhyML (Guindon et al., 2010) was used to obtain a maximum‐likelihood tree using the default model of HKY85 + G. Trees were visualized using FigTree version 1.4.2 (http://tree.bio.ed.ac.uk/software/figtree/).

The expansin sequences from (Li et al., 2003) were downloaded from the National Center for Biotechnology Information (NCBI) Web site (Coordinators, 2017). The accession numbers are as follows: AF247163.1, AF247164.1, AF247165.1, AF394545.1, AF394546.1, AF394548.1, AF394549.1, AF394550.1, AF394551.1, AF394552.1, AF394553.1, AF394554.1, AF394555.1, AF394556.1, AF394557.1, AF394558.1, AF394559.1, AF394560.1, AF394561.1, AF394562.1, U30477.1, U30479.1, U85246.1, Y07782.1, AF261270.1, AF391105.1, AF391106.1, AF391108.1, AF391110.1, AF391111.1, AF261271.1, AF261272.1, AF261273.1, AF261274.1, AF261275.1, AF261276.2, AF261277.1, AF261278.1, AF466188.1, AY039023.1, AY046928.1, AY046929.1, AY100692.1, AY100693.1, AY100694.1, and U95968.1.

The ribonuclease accession numbers are as follows: AAF82615.1, AAG09465.1, gi|15149819, ref|XP_002311302.1, ref|XP_002311303.1, ref|XP_002316136.1, ref|XP_002321228.1, gb|EEE95823.2, gb|AAM18521.1, gb|AAS01727.1, gb|AAS07016.1, gi|68563425, gi|3860325, ref|XP_001691379.1, gi|113374061, gb|AAB58718.1, gb|AAB58719.1, gb|AAF45043.1, gi|71611076, gb|AAF45022.1, gb|AAM80567.1, gi|133173, gi|31620998, gi|31621000, gi|31621002, gi|116058653, gb|AAA21135.1, gi|976231, gi|976233, gi|18394083, gi|18394085, gi|18396065, gi|18405157, gi|1710615, gi|1710616, gi|116634825, gi|50513542, gi|7707689, gi|1698670, gi|195628852, gi|642956, gb|AAC49326.1, Et_s3159‐0.24‐1, LOC_Os07g43670.1, LOC_Os01g67180.1, LOC_Os08g33710.1, LOC_Os09g36680.1, LOC_Os09g36700.1, LOC_Os01g67190.1, Os07g0629900, Obart07g23070, Orgla07g0180700, Oglum07g23130, Oniva07g22690, Bgiosga026191, Orufi07g24240, Os07g0629300, Opunc07g21760, Obart07g23080, Oglum07g23140, Oniva11g11010, Bgiosga026192, and Orufi07g24250.

### 2.18 Accession numbers

This project has been archived at GenBank under BioProject PRJNA413657 with BioSamples: GNY1: SAMN07764637, GNY3:SAMN07764639, GNY10:SAMN07764640, and GNY12: SAMN07764642. The reads have been deposited in the GenBank Sequence Read Archive as study SRP119988 with accession numbers: SRR6175533 (GNY1‐1), SRR6175534 (GNY2‐1), SRR6175535 (GNY3‐1), SRR6175536 (GNY1‐2), SRR6175529 (GNY2‐2), SRR6175530 (GNY3‐2), SRR6175531 (GNY10), SRR6175532 (GNY11), and SRR6175528 (GNY12).

## 3 RESULTS

### 3.1 Time series on growth of tef shoots under early waterlogging

The time course Experiment 1 was undertaken to measure shoot growth of *Alba*,* Quncho*, and *Tsedey* genotypes under waterlogging and normal watering conditions where plant height was measured at the interval of 2–4 days. The difference between the height of the control plants and those treated with waterlogging varied between the three cultivars in response to waterlogging (Fig. S1). For *Alba*, the difference between the height of the control plants and those that were waterlogged was not significantly different at any time (Mann–Whitney *U*‐test, *p* ≤ .05). The waterlogged *Quncho* plants were significantly shorter than the control plants at 3 and 7 days after watering, while the waterlogged *Tsedey* plants were significantly taller than the control plants 1, 3, 4, and 7 days after the onset of stress. At 8 days after waterlogging treatment, the heights of the waterlogged and control plants were no longer significantly different for any cultivar.

### 3.2 Time series studies on plant development under early waterlogging

Experiment 2 is a repetition of Experiment 1 but with four waterlogged and four control plants harvested at 2, 4, 7, 9, 11, 16, and 24 days after the waterlogging stress was applied. This enabled visualization of root characteristics as well as measurements of root and shoot biomass. The *Quncho* and *Tsedey* varieties were chosen for this experiment as their responses to waterlogging in Experiment 1 differed, namely *Tsedey* grew taller during the onset of waterlogging, while *Quncho's* growth was suppressed.

**Figure 1:**
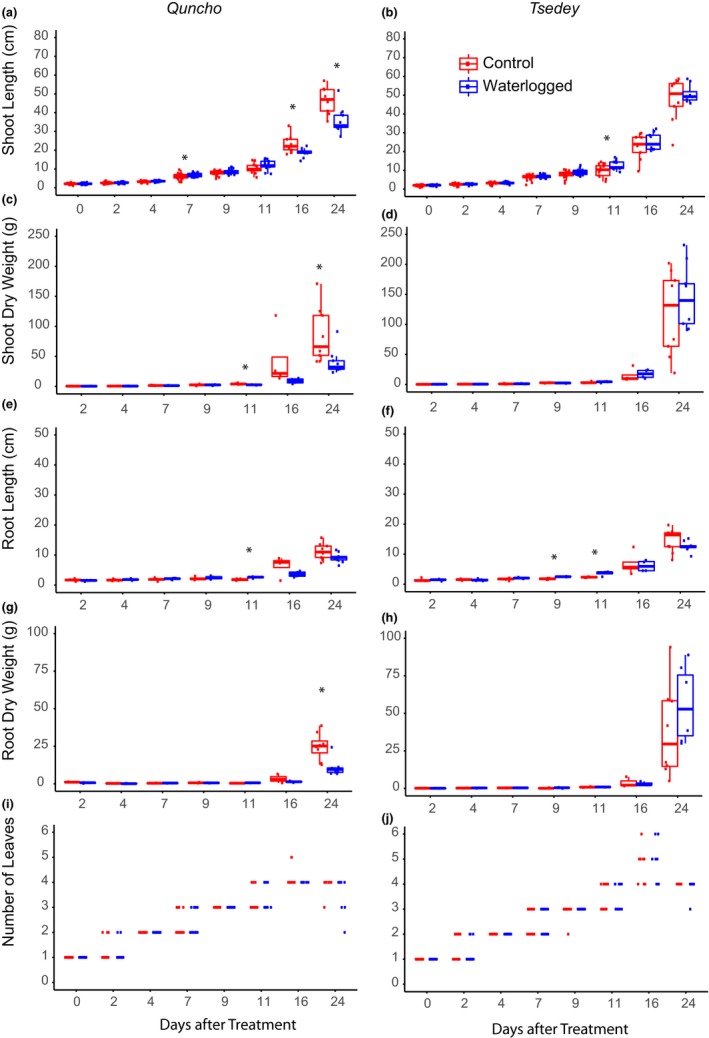
Growth of 4‐day‐old seedlings of two tef genotypes (*Quncho* and *Tsedey*) subjected to waterlogging and normal watering. Shoot length (a, b), shoot dry weight per plant (c, d), root length (e, f), root dry weight per plant (g, h), and number of leaves per plant (i, j). Means marked with an asterisk are significantly different from each other (Mann–Whitney: *p ≤ .05)

At each time point, shoot dry weight, root dry weight, root length and shoot length, and the number of leaves were measured (Figure 1 for all days, Table 2 for day 24, and all raw data in Tables S1 (*Quncho*) and S2 (*Tsedey*)). As in Experiment 1, *Tsedey* grew more robustly than *Quncho* in the waterlogged environment. The number of replicates in Experiment 2 (three or four replicates) was smaller than that of Experiment 1 (nine replicates) because of the destructive sampling. The small number of replicates combined with the large variance between observations resulted in fewer statistically significant measurements in Experiment 2. On the last day, day 24, all remaining plants were harvested, and their parameters measured. For this reason, there were nine replicates of each condition and genotype on day 24.

**Table 2:** Growth characteristics of *Alba, Quncho*, and *Tsedey* after 24 days of waterlogging at the early growth stage (4 days)

Parameter	*Quncho*			*Tsedey*		
	Control	Waterlog	% of control	Control	Waterlog	% of control
Shoot length	46.5 ± 7	35.8 ± 7.1	76.9*	47.6 ± 11.6	50.6 ± 4.8	106
Shoot dry weight	82.6 ± 48	40.4 ± 20	48.9*	118 ± 68	145.2 ± 52	123
Root length	11.2 ± 2.7	9.1 ± 1.6	81.3	14.5 ± 3.8	12.6 ± 1.7	87
Root dry weight	24.8 ± 9	10.8 ± 5.7	43.5*	38.0 ± 31	56.1 ± 24	148
Number of leaves	3.9 ± 0.4	3.4 ± 0.8	87.2	4.0 ± 0	3.9 ± 0.4	97.5

Four‐day‐old plants were grown in soil either with normal watering or with water maintained at 1 cm below the soil surface. All values are means (*n* = 3 or 4) ± SD. Significance differences at *p* ≤ .05 are denoted with a * (Mann–Whitney).

#### 3.2.1 Shoot growth

In the *Tsedey* cultivar, the shoot length, measured from the base of the plant to the end of the longest leaf, was in most cases similar in the control and waterlogged plants. However, the waterlogged plants were significantly longer than those of the control plants at 11 days after stress (Figure 1). The shoot dry weight was similar for *Tsedey* plants under control and waterlogged conditions.

For *Quncho*, the length of the shoot of the waterlogged plants was significantly smaller than that of the control plants at 7, 16, and 24 days after stress. The waterlogged plants started showing signs of stress (smaller plants with browning and dying leaves) at 16 days after the onset of stress. This resulted in the shoot dry weight of *Quncho* being significantly lower at 11 and 24 days after stress.

The summary of growth statistics at 24 days after waterlogging is shown in Table 2. After 24 days of waterlogging, *Quncho* subjected to waterlogging had 49% less shoot dry weight, while the shoot dry weight of *Tsedey* increased by 23%. In addition, the shoot length of *Quncho* was negatively affected, while *Tsedey* consistently grew larger under waterlogged conditions. The number of leaves did not change significantly for either cultivar.

#### 3.2.2 Root growth

The root structure of tef plants contains many fine root hairs. As the plants were grown in soil, it was necessary to wash the root ball before drying and measurement. To remove all soil without damaging, the fine root structure was difficult and added uncertainty to the measurements of root weight. In terms of root length, the roots of *Tsedey* subjected to early waterlogging were significantly longer at 9 and 11 days after stress, while the roots of *Quncho* subjected to early waterlogging were significantly shorter at 11 days after treatment (Figure 1). Similarly, *Quncho* roots had significantly less dry weight at 24 days after waterlogging where they were reduced by 56% compared to that of *Tsedey* which had increased in weight by 48% (Table 2). The length of the longest root of both *Tsedey* and *Quncho* decreased under waterlogging by about 20% (roots taken from Experiment 2). However, it was observed that under waterlogging, the fine roots of the control plants were replaced with significantly shorter, thicker, and whiter adventitious roots in all cultivars studied.

### 3.3 Root growth of cereal plants waterlogged from 4 days

For better visualization of the roots, in Experiment 3, single plants of *Alba*,* Quncho*, and *Tsedey* were grown in rhizotrons to view the root growth, and indeed, after harvesting, the root ball of *Tsedey* was sometimes much larger under waterlogging than under control conditions. However, the variation in root growth between plants and experiments was high.

For comparison, the experiment was repeated with *Alba, Tsedey*, wheat, and maize (Experiment 4). In *Tsedey*, the control roots were longer, but the waterlogged plants had thicker roots and more adventitious roots than normally watered plants (Fig. S2). The aboveground portion of the control and waterlogged *Tsedey* plants looked similar (not shown). The root plate of the waterlogged *Tsedey* plants had a wider root plate, while the roots of the control plants tended to grow longer and toward the bottom of the pot. While the *Alba* control plants had more roots at every stage than the waterlogged roots, clear adventitious root growth was not visible (not shown). The waterlogged plants were always smaller and had fewer leaves and tillers.

In wheat, after one day of waterlogging, the root systems of the control and waterlogging plants were similar (Fig. S3). However, after 3 days of waterlogging, the control plants had distinctly more roots than the waterlogged plants, and this phenomenon became more pronounced with time. Both control and waterlogged plants grew adventitious roots, but they were more pronounced in the control plants. By 25 days, the control roots filled the rhizotron and the plants looked much healthier than the waterlogged.

In maize, waterlogging was started on plants 7 days after germinated seeds were transplanted into rhizotrons. The difference in root growth becomes apparent one week after waterlogging where the roots of the control plants were longer (Fig. S4) and the roots of the waterlogged plants thicker. After 10 days, the waterlogged roots formed a fine root structure but remained in the upper part of the rhizotron, while the roots of the control plant reached the bottom. At 25 days, the maize roots of the control plant filled the rhizotron.

### 3.4 Physiological response of tef to waterlogging at the tillering stage

**Figure 2:**
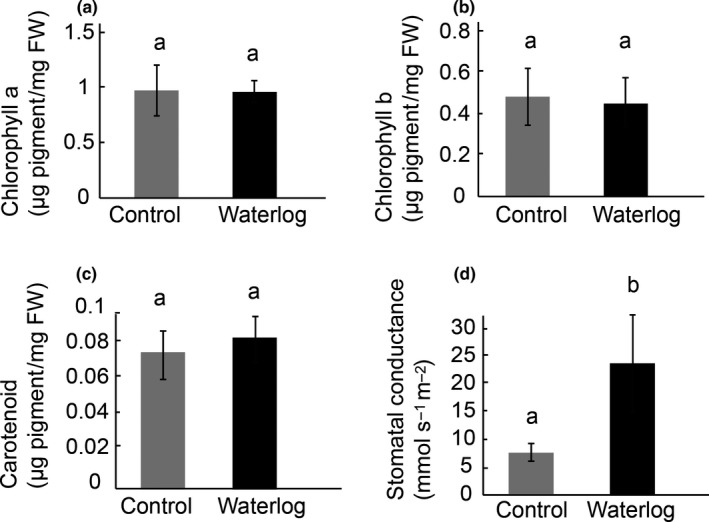
Physiological response of tef plants to waterlogging. The amounts of chlorophyll a (a), chlorophyll b (b), and carotenoid (c), as well as the stomatal conductance (d), were measured for waterlogged and control plants. Treatments marked with different letters have values that are significantly different (Mann–Whitney, *p* ≤ .05)

Nineteen‐day‐old seedlings of improved tef variety *Tsedey* (DZ‐Cr‐37) were exposed to either normal watering or waterlogging for 9 days. Physiological measurements of stomatal conductance, as well as carotenoid, chlorophyll a, and chlorophyll b, were made before the plants were harvested and the tissue sent for sequencing (Experiment 5). Only stomatal conductance was altered significantly under waterlogging conditions (Figure 2). The difference was large, however, as stomatal conductance was roughly three times higher under waterlogging conditions than controls.

### 3.5 Aerenchyma formation in roots under waterlogging at the tillering stage

**Figure 3:**
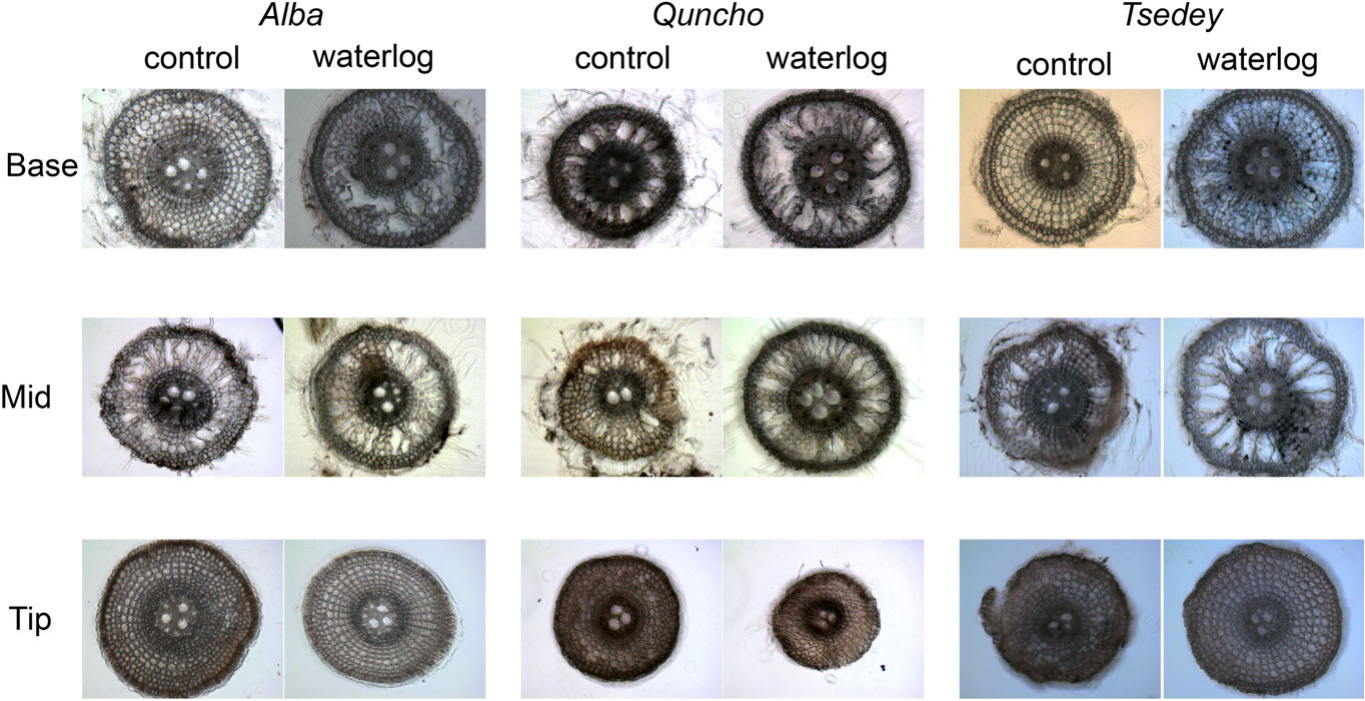
Formation of aerenchyma in adventitious roots of three tef genotypes at the tillering stage. Unstained cross sections of base, middle, and tip of roots of *Alba, Tsedey*, and *Quncho* genotypes subjected to 9 days of waterlogging at the tillering stage. All pictures are on the same scale. Scale bar: 100 μm

**Figure 4:**
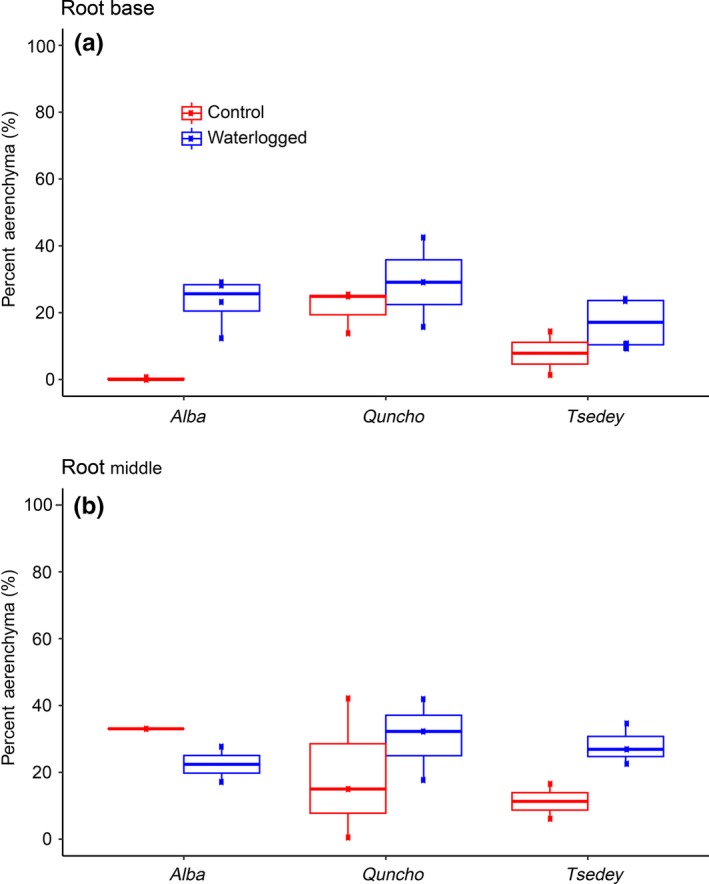
Percentage of cortex occupied by aerenchyma in three cultivars under control and waterlogged conditions. The percentage of aerenchyma in *Alba*,* Quncho*, and *Tsedey* was measured at the root base (a) and middle (b) under control and waterlogged conditions. No aerenchyma were present in the root tip

Experiment 6 was conducted to observe aerenchyma formation at the tillering stage, 19‐day‐old seedling subjected to 9 days of waterlogging or normal watering. Cross sections of extracted adventitious roots were taken to observe whether aerenchyma were being formed (Figure 3: summary, Fig. S5: all photographs). The number of roots successfully cross‐sectioned ranged from 1 to 3 per genotype and condition. The percentage of cortex area containing aerenchyma in the base, middle, and tip of the root is summarized in Figure 4. All three cultivars had some aerenchyma under normal watering conditions with the number of aerenchyma increasing under waterlogging. The middle of the roots contained more aerenchyma than the base of the root, while no aerenchyma were observed in the root tips of any cultivar under either waterlogged or control condition. *Quncho* and *Tsedey* exhibited constitutive aerenchyma, the size of which increased dramatically upon waterlogging. *Alba* had little constitutive aerenchyma at the base but was able to form them upon excessive moisture stress.

In addition, the number of adventitious roots was counted for replicates of this experiment. For all three genotypes, the number of adventitious roots increased with waterlogging. The variance was high, however, from 109% in *Alba* to 336% in *Tsedey* (Table S3).

The roots of one replicate from each day of Experiment 2 (waterlogging of 4‐day‐old plants for 22 days) were used to visualize the formation of aerenchyma of *Quncho* and *Tsedey* with samples taken at 2, 4, 7, 9, 16, and 24 days after waterlogging (Fig. S6). *Quncho* was the earliest to show aerenchyma formation which appeared only 4 days after waterlogging, while in *Tsedey* they were first observed 7 days after stress.

### 3.6 RNA‐Seq study of differential expression at the tillering stage

Changes in gene expression in leaves of the *Tsedey* genotype (Experiment 7) were measured using RNA‐Seq (pipeline is shown in Fig. S7). For transcriptome sequencing, a total of 132,416,127 sequence reads were generated with Illumina sequencing (Table 3). These reads were filtered for adaptor and vector sequences and trimmed such that the Phred score was greater than 30 across the sequence.

**Table 3:** RNA‐Seq data collected and mapping statistics

Treatments	Library	Length after trimming (bp)	Number of reads	Number of bases after trimming (bp)	Percentage uniquely mapped (%)	Percentage of reads mapped to multiple loci	Percentage unmapped
Normal watering	GNY1	70	6,817,359	477,215,120	67.5	18.5	14.0
	GNY1b	90	18,854,229	1,696,880,610	70.6	15.2	14.2
	GNY10	90	35,853,151	3,226,783,590	75.3	19.2	5.5
	Subtotal		61,524,739	5,400,879,320			
Waterlogging	GNY3	70	9,413,900	658,973,000	68.3	15.6	14.6
	GNY3b	90	23,625,313	2,126,278,170	71.7	15.4	12.9
	GNY12	90	37,852,175	3,406,695,750	75.1	19.3	5.5
	Subtotal		70,891,388	6,191,946,920			
	Total		132,416,127	11,592,826,240			

#### 3.6.1 Annotation of the background set using Blast2GOand Mercator

Although the Tef Genome Project provided annotations, only a small percentage of sequences had gene ontology (GO) annotations, which are helpful for enrichment analysis. Therefore, the background genes were reannotated with descriptions and GO terms, InterPro protein signatures (Finn et al., 2017), and KEGG pathways (Kanehisa, Furumichi, Tanabe, Sato, & Morishima, 2017) using Blast2GO (Conesa & Götz, 2008). These annotations are available in Table S4 and at http://www.tef-research.org/genome.html. For the background set, the top hit was from *Setaria italica* for 14,777 proteins followed by *Sorghum bicolor* (5,436 proteins), *Zea mays* (4,571 proteins), and *Oryza sativa* (2,771 proteins).

GO terms were assigned via Blast2GO using the GOSlim plant annotations resulting in 27,338 proteins annotated with GO terms in the background set (Fig. S8). For the biologic process, the GO terms “metabolic process,” “cellular process,” “single‐organism process,” “biologic regulation,” and “response to stimulus” were the five most represented categories. The molecular function category was dominated by “catalytic activity” and “binding” with “transporter activity” also being well represented. The cellular components most often found were “cell,” “organelle,” and “membrane”.

In addition, the background set was annotated with pathways using the KEGG annotation system in Blast2GO. Prediction of the pathways containing the background proteins resulted in 127 pathways containing 8,768 proteins (25.2% of the genes detected in the experiment were classified). The pathways including the most transcripts were “purine metabolism,” “starch and sucrose metabolism,” “phenylpropanoid biosynthesis,” “phenylalanine metabolism,” “pyrimidine metabolism,” and “glycolysis and glucogenesis.”

#### 3.6.2 Annotation of the background set using Mercator

Mercator, a Web application that assigns DNA or protein sequences to one of 35 MapMan bins (Lohse et al., 2014), was also used for annotation of the background set. MapMan categorization provides an overview of metabolism and cellular process and is tailored for functional annotation of plants (Thimm et al., 2004). Many of these categories deal with metabolic pathways and enzyme functions, providing an overview of the metabolic networks involved in the response. The results of the mapping of the background set revealed the categories: transport (11.7%), cell wall (10.0%), protein (8.3%), and cell (8.3%) to be the most represented functional categories (Fig. S9).

#### 3.6.3 Differential expression

Differential expression analysis was performed to determine the change in transcript expression from the waterlogging treatment to the control (normal watering) (Fig. S7). First, the reads were mapped to the genome using STAR aligner (Dobin et al., 2013). A count table was generated for each predicted gene from the tef genome for each sequencing dataset using HTSeq (Anders et al., 2015), which was given the genome and the predicted locations of the genes provided by the tef genome sequencing (Cannarozzi et al., 2014). Only the roughly 70% of the reads that map uniquely to one location in the genome were used for counting (Table 3). Count tables generated by HTSeq were then used as input into DESeq (Anders & Huber, 2010). Differentially expressed genes were defined as those with an adjusted *p*‐value of ≤.05 and with a fold change in expression greater than two and are tabulated in Tables 4, 5, and S5. Downregulation was defined as fewer transcripts in the treatment compared to control and the opposite for upregulated. Differential expression analysis identified 37 genes significantly upregulated and 19 genes significantly downregulated under waterlogging conditions. Tables 4 and 5 containing the differentially regulated genes are listed in the MapMan category number.

**Table 4:** Genes upregulated under flooding, their Blast2GO and MapMan annotations, their fold change, and *p*‐value organized using the MapMan categories

Name	Blast2GO description	Mercator function	Fold change	Log2 fold change	Adj. *p*‐value^a^
Photosynthesis					
Et_s1919‐1.19	Ribulose bisphosphate carboxylase oxygenase activase chloroplast‐like RuBisCO	1.3.13 PS.calvin cycle.rubisco interacting	8.33	3.06	.0178
Major carbohydrate metabolism					
Et_s6672‐0.28	Granule‐bound starch synthase ii	2.1.2.2 major CHO metabolism.synthesis.starch.starch synthase	11.34	3.50	.0001
Et_s66‐0.13	Granule‐bound starch synthase ii	2.1.2.2 major CHO metabolism.synthesis.starch.starch synthase	5.23	2.39	.0231
Et_s2233‐0.29	Glucose‐6‐phosphate phosphate translocator precursor	2.2.2.1.2 major CHO metabolism.degradation.starch.starch cleavage.β amylase	10.57	3.40	.0002
Et_s2217‐0.39	β‐amylase	2.2.2.1.2 major CHO metabolism.degradation.starch.starch cleavage.Β‐ amylase	15.70	3.97	
Et_s7847‐0.25	NADP‐dependent malic enzyme	8.2.10 TCA/org transformation.other organic acid transformatons.malic’	6.46	2.69	.0042
Growth					
Et_s2486‐1.7	β‐expansin 1a precursor	10.7 cell wall.modification	11.60	3.54	.0001
Et_s5792‐0.0	β‐expansin 1a precursor	10.7 cell wall.modification	8.58	3.10	.0231
Et_s5823‐0.14	Xyloglucan endotransglucosylase hydrolase protein 8 precursor	10.6.2 cell wall.degradation.mannan‐xylose‐arabinose‐fucose 10.7 cell wall.modification	6.01	2.59	.0171
Et_s9915‐0.7	β‐expansin 1a isoform ×1	10.7 cell wall.modification	5.90	2.56	.0304
Et_s11804‐0.16	Probable xyloglucan endotransglucosylase hydrolase‐like	10.7 cell wall.modification	6.42	2.68	.0108
Metal handling and acquisition					
Et_s12869‐0.19	Ferric reduction oxidase chloroplast‐like	15.1 metal handling.acquisition	6.46	2.69	.0068
Et_s13065‐0.28	Ferric reduction oxidase chloroplast‐like	15.1 metal handling.acquisition	6.24	2.64	.0444
Et_s1056‐0.47	Heavy metal‐associated domain‐containing expressed	“35.2” “not assigned.unknown”	12.43	3.64	.0009
Secondary metabolism					
Et_C8513699‐0.0	Transresveratrol di‐o‐methyltransferase‐like	16.2 secondary metabolism.phenylpropanoids	8.81	3.14	.0311
Et_s20148‐0.9	Cinnamoyl reductase 1‐like	16.2.1.7 secondary metabolism.phenylpropanoids.lignin biosynthesis.CCR1	17.5176	4.13	.0132
Et_s517‐0.16	Phytoene synthase 2	16.1.4.1 secondary metabolism.isoprenoids.carotenoids.phytoene synthase	6.73	2.75	.0311
Et_s4037‐1.43	GDP‐l‐galactose phosphorylase 2‐like	“21.2.1.2” “redox.ascorbate and glutathione.ascorbate.GDP‐L‐galactose‐hexose‐1‐phosphate guanyltransferase”	5.94	2.57	.0077
Et_s786‐1.34	PAP‐specific phosphatase mitochondrial‐like	“23.2” “nucleotide metabolism.degradation” “et_s786‐1.34‐1”	7.08	2.82	.0328
Et_s4382‐0.38	Short‐chain dehydrogenase tic chloroplast‐like	“26.22” “misc.short chain dehydrogenase/reductase (SDR)”	11.18	3.48	.0077
RNA regulation					
Et_s7183‐0.17	Salt tolerance‐like protein	“27.3.7” “RNA.regulation of transcription.C2C2(Zn) CO‐like, Constans‐like zinc finger family”	7.52	2.91	.0011
Et_s6551‐0.36	Salt tolerance‐like protein	“27.3.7” “RNA.regulation of transcription.C2C2(Zn) CO‐like, Constans‐like zinc finger family”	5.61	2.49	.0180
Protein functions					
Et_s3682‐2.26	Pentatricopeptide repeat‐containing protein at5 g25630‐like	“29.4” “protein.postranslational modification”	7.77	2.96	.0055
Et_s409‐2.7	Carboxyl terminal‐processing peptidase chloroplastic	“29.5.5” “protein.degradation.serine protease”	8.72	3.12	.0062
Et_s2871‐0.32	C‐terminal processing chloroplastic‐like	“29.5.5” “protein.degradation.serine protease”	6.09	2.61	.0399
Et_s1636‐1.49	BTB POZ and TAZ domain‐containing protein 3 isoform ×1	“29.5.11.4.5.2” “protein.degradation.ubiquitin.E3.BTB/POZ Cullin3.BTB/POZ”	7.17	2.84	.0399
Signaling					
Et_s781‐0.29	Early light‐induced protein	“30.11” “signalling.light”	10.54	3.40	.0077
Cell division					
Et_s10318‐0.21		“31.2” “cell.division”	10.56	3.41	.0018
Et_s7686‐1.48		“31.2” “cell.division” Regulator of chromosome condensation (RCC1) family protein;	8.67	3.12	.0075
Transport					
Et_s7784‐0.11	Magnesium proton exchanger isoform 1	“34.12” “transport.metal”	21.45	4.42	.0055
Et_s6198‐0.9	High‐affinity nitrate transporter‐like	“34.4” “transport.nitrate” “et_s6198‐0.9‐1”	12.43	3.64	.0149
Et_s594‐1.9	High‐affinity nitrate transporter‐like	“34.4” “transport.nitrate”	10.09	3.33	.0221
Unknown					
Et_s90‐1.51		“35.2” “not assigned.unknown”	29.46	4.88	.0098
Et_s304‐1.36	Late embryogenesis abundant protein lea5‐d‐like	“35.2” “not assigned.unknown”	6.70	2.74	.0106
Et_s1487‐0.5		“35.2” “not assigned.unknown”	8.55	3.10	.0355
Et_s2486‐1.2	β‐expansin 1a precursor	“not assigned.unknown” “et_s2486‐1.2‐1”	6.4651	2.69	.0373
Et_s1663‐0.25		“35.2” “not assigned.unknown”	5.33	2.41	.0378

a Adjusted *p*‐value from HTSeq.

**Table 5:** Genes downregulated under flooding, their Blast2GO and MapMan annotations, their fold change, and *p*‐value organized using the MapMan categories

Name	Blast2GO description	Mercator function	Fold change	Log2 fold change	Adj. *p*‐value^a^
Et_s2617‐0.51	Aldo‐keto reductase family 4 member c9	“3.5” “minor CHO metabolism.others”	0.190	−2.39	.0493
Et_s1215‐0.33	Malate glyoxysomal	“6.2” “gluconeogenesis/glyoxylate cycle.malate synthase”	0.106	−3.23	.0089
Cofactor and vitamin metabolism					
Et_s4931‐1.11	Phosphomethylpyrimidine chloroplastic‐like isoform ×1	“18.2” “Co‐factor and vitamine metabolism.thiamine”	0.078	−3.67	.0082
Et_s780‐1.30	Thiazole biosynthetic enzyme thi4 family	“18.2” “Co‐factor and vitamine metabolism.thiamine”	0.124	−3.01	.0231
Abiotic stress					
Et_s346‐4.13	Bifunctional nuclease 2‐like isoform ×1	“20.2.4” “stress.abiotic.touch/wounding”	0.022	−5.49	.0000
Et_s11116‐0.0	Chaperone protein 1‐like	“20.2.1” “stress.abiotic.heat”	0.016	−5.90	.0000
Et_s3654‐1.34	Bifunctional nuclease 2‐like isoform ×1	“20.2.4’ “stress.abiotic.touch/wounding”	0.072	−3.79	.0007
Et_s3572‐0.37	Thioredoxin‐like 3‐chloroplastic	“21.1” “redox.thioredoxin”	0.080	−3.63	.0004
Et_s190‐0.32	Class III peroxidase	“26.9” “misc.glutathione S transferases”	0.116	−3.11	.0011
Regulation/signaling					
Et_s2666‐0.25	TUBBY‐like f‐box protein 7‐like	“27.3” “RNA.regulation of transcription”	0.133	−2.91	.0020
Et_s3159‐0.24	RNase s‐like protein precursor	“27.1.19” “RNA.processing.:qucleases”	0.090	−3.47	.0399
Et_s521‐0.23	GTP‐binding protein hflx	“30.5” “signalling.G‐proteins”	0.084	−3.57	.0009
Et_s788‐0.23		“31.1” “cell.organisation”	0.032	−4.94	.0000
Et_s4931‐1.10		“31.1” “cell.organisation”	0.029	−5.09	.0009
Transport					
Et_s819‐0.0	Aquaporin tip4‐2	“34.19.2” “transport.Major Intrinsic Proteins.TIP”	0.133	−2.90	.0088
Et_s4179‐0.34	Aquaporin tip4‐1	“34.19.2” “transport.Major Intrinsic Proteins.TIP”	0.146	−2.77	.0053
Unknown					
Et_s5368‐0.25	Single‐strand binding protein	“35.2” “not assigned.unknown”	0.084	−3.56	.0328
Et_s482‐0.32		“35.2” “not assigned.unknown”	0.085	−3.55	.0387

a Adjusted *p*‐value from HTSeq.

#### 3.6.4 Functional classification of genes differentially expressed under waterlogging stress

Mercator was also used to classify the differentially regulated sequences into the 35 MapMan functional plant categories (Fig. S10) (Thimm et al., 2004). Under waterlogging, the upregulated categories included bin 10: cell wall (15.8%), bin 29: protein (10.5%), bin 34: transport (10.5%), bin 2: major carbohydrate metabolism (7.9%), and bin 16: secondary metabolism (7.9%) with 15.8% unassigned. The downregulated categories included bin 20: stress (13.64%), bin 31: cell (13.64%), bin 34: transport (13.64%), bin 27: RNA (9.1%), bin 18: cofactor and vitamin metabolism (9.1%), bin 6: glyconeogenesis (4.6%), bin 3: minor CHO metabolism (4.55%), bin 21: redox (4.55%), and bin 30: signaling (4.55%) with the classification of 9.1% as unassigned.

### 3.7 Validation of the RNA‐Seq expression values

**Figure 5:**
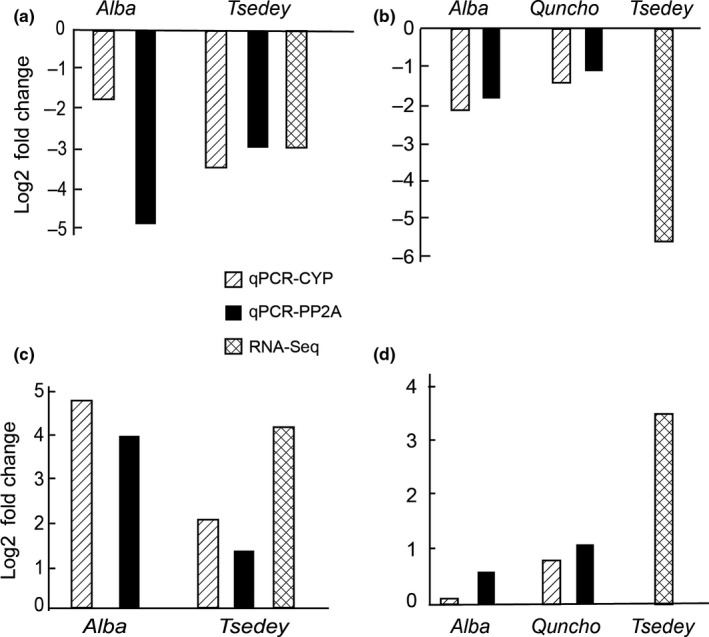
Validation of RNA‐Seq expression measurements by qPCR in three tef genotypes. Aquaporin (a), bifunctional nuclease 2‐like gene (b), cinnamoyl reductase‐like gene (c), and granule‐bound starch synthase (d) were regulated under waterlogging conditions using RNA‐Seq. Expression measurements for these four transcripts were verified with qPCR, using either CYP or PP2A as reference genes. Three replicates were used for each genotype, condition, and transcript

From the genes determined to be significantly differentially expressed under waterlogging conditions using RNAS‐Seq on the *Tsedey* genotype, four genes were chosen for experimental validation with qPCR using *Alba, Quncho,* and *Tsedey* tef genotypes. These include two genes that were upregulated, a cinnamoyl reductase‐like gene and a granule‐bound starch synthase gene, as well as two that were downregulated, a bifunctional nuclease 2‐like gene and an aquaporin. Two genes, serine/threonine–protein phosphatase (PP2A) and cyclophilin/peptidyl‐prolyl Isomerase (CYP), have been identified as the most stable for use as reference genes in qPCR of abiotic stress response genes in sorghum (Reddy et al., 2016). All measurements were taken using both reference genes. Comparisons between the RNA‐Seq expression measurements in *Tsedey* and the qPCR expression in *Tsedey*,* Alba*, and *Quncho* are shown in Figure 5 and Table S6. All four genes were regulated in the same direction with similar magnitudes. The use of the two reference genes resulted in similar behavior except in the *Alba* genotype for the aquaporin gene and for granule‐bound starch synthase (Figure 5).

## 4 DISCUSSION

Eight experiments were carried out to probe the morphology of roots, growth of shoots and roots, and gene expression. The experiments were carried out either on 4‐day‐old seedlings with parameter measurement every 2–4 days for up to 24 days (early waterlogging) or on plants at the tillering stage (19‐day‐old plants which were then waterlogged for 9 days).

### 4.1 Growth

The *Tsedey* genotype grew faster under early waterlogging conditions, while the growth of *Quncho* was suppressed (Figure 1 and S1). Measurements of *Tsedey* seedling growth over time showed that significant differences in growth occurred in the first week of waterlogging. After 22 days of waterlogging, the shoot dry weight of *Tsedey* was 123% larger under waterlogging, while for *Quncho* it was reduced to 49% of the control length (Table 2). The largest difference between *Tsedey* and *Quncho* tef genotypes was seen in the roots, where *Tsedey* consistently grew a larger root mass when waterlogged. The root length decreased for both genotypes, but the number of adventitious roots increased for both, resulting in a denser root mass. The root dry weight was significantly increased in *Tsedey* by 148%, while in *Quncho* it was reduced to 48%. A part of the difference may be attributed to the fact that thicker roots are less likely to be damaged or removed when washing the root ball. In a similar study, the dry weight of the flooding‐tolerant *Zea nicaraguensis* decreased by 54%, while in the flooding‐susceptible *Zea mays*, the dry weight was reduced by 60% (Abiko et al., 2012). A global increase in biomass under waterlogging conditions is often associated with waterlogging adaptation (Naidoo & Naidoo, 2015) as is the ability to produce adventitious roots (Steffens, Wang, & Sauter, 2006). The number of leaves was not affected, but the leaves of *Quncho* started browning after 16 days, a sign of waterlogging stress.

### 4.2 Aerenchyma formation at the tillering stage

Cross sections of three tef genotypes (*Alba*,* Quncho*, and *Tsedey*) revealed the amount of aerenchyma in the root tip, middle, and base of the roots under conditions of waterlogging and normal watering (Figure 3 and Fig. S5). No genotype formed aerenchyma in the root tips under any condition, as was shown for *Z. mays* (Abiko et al., 2012). *Alba* had aerenchyma only in the middle of the root under control conditions where after 9 days of waterlogging, aerenchyma were induced in the base and the number and size of aerenchyma in the middle of the root increased. *Quncho* had more and larger constitutive aerenchyma than *Tsedey*, especially at the base of the root. However, both cultivars developed larger aerenchyma upon waterlogging in both the middle and the base of the root. In a similar study, *Zea mays* roots formed a negligible amount of aerenchyma in drained soil which increased to 15% by area under waterlogging. The roots of *Z. nicaraguensis*, a waterlogging‐tolerant species, had 22% aerenchyma by area, which increased to 29% under waterlogging (Abiko et al., 2012). Aerenchyma are induced by waterlogging but are found constitutively in wetland or waterlogging‐adapted species (Abiko et al., 2012; Justin & Armstrong, 1987). A QTL for aerenchyma formation has been identified in barley, and the newly developed markers explain 44% of the phenotypic variance (Zhang et al., 2016), while a recently discovered allele in wild barley is said to account for 76.8% of the phenotypic variance (Zhang et al., 2017).

### 4.3 Physiological response at the tillering stage

Measurements of the seedling growth over time show that the largest changes in growth occurred directly after the application of the stress, while in this experiment the tissue was collected 9 days after the application of the stress. For the physiological measurements, no significant difference was found in chlorophyll a, chlorophyll b, or carotenoid which may indicate that the plants were not stressed to a large extent or had already adapted to the stress (Figure 2). Stomatal conductance, however, increased threefold under flooding. Caudle and Maricle suggested that the characteristics of plants that correlated best with tolerance to flooding were photosynthesis rate, respiration upon flooding, and the ability to avoid oxygen shortage (Caudle & Maricle, 2012). Leaf‐level stomatal conductance was also suggested to be an indicator of flooding tolerance in comparison with the responses of the flooding‐sensitive Johnson grass (*Sorghum halepense*) and the flooding‐tolerant common reed (*Phragmites australis*). In the sensitive *Sorghum halepense*, transpiration decreased in response to flooding, while in the tolerant *Phragmites australis*, the opposite occurred (Waring & Maricle, 2013). A study in tolerant versus sensitive wheat concluded that a significant reduction in grain and straw yield happened only if the waterlogging was prolonged for more than 20 days (Arduini, Orlandi, Pampana, & Masoni, 2016).

### 4.4 Differentially expressed genes under waterlogging

A total of 56 genes were found to be regulated under waterlogging at the tillering stage (Tables 4, 5 and S5). The small number of genes for which significant changes in expression were found between normal and waterlogging conditions in *Tsedey* contrasts with studies in *Brassica napus L*. roots where 4432 genes changed expression after 12 h of waterlogging (Zou et al., 2013), while in *Jatropha* 1968 mRNA transcripts had a significant change in abundance (Juntawong et al., 2014). Similarly, in cypress tree (*Taxodium “Zhongshansa 406”*), 2090 differentially expressed genes were found in roots, while only 394 were found in shoots (with 174 shared between the two groups). The RNA used in the current study was extracted from seedlings (not including the roots) 9 days after waterlogging at the tillering stage, which may explain the relatively small number of genes detected.

The differentially regulated genes are ordered by their MapMan annotation bins in Tables 4 and 5 and are discussed in this order. The distribution of the functional categories of the regulated genes shows that after 19 days of waterlogging, the MapMan bins with the most upregulated genes were 15.8% cell wall, 11% transport, 11% protein (amino acid activation, synthesis, targeting, modification, and degradation), major carbohydrate metabolism, and secondary metabolism. The most downregulated bins were as follows: 14% cell (cell organization, division, cycle, and vesicle transport), 14% stress, 14% transport, 9% miscellaneous, 9% RNA, and 9% cofactor/vitamin metabolism (Fig. S10).

#### 4.4.1 Photosynthesis

In the expression experiments, RuBisCO activase (ribulose bisphosphate carboxylase oxygenase activase), Et_s919‐1.19, was upregulated under flooding conditions in *Tsedey*. RuBisCO is important in the first step of fixing carbon dioxide into organic carbon molecules in the Calvin cycle. The principal role of RuBisCO activase is to release inhibitory sugar phosphates from the active sites of RuBisCO (Jordan & Chollet, 1983) and it also acts as a chaperone during stress (Rokka, Zhang, & Aro, 2001). In tomato, RuBisCO activase has been found to decrease under flooding stress and is susceptible to degradation from reactive oxygen species (Ahsan et al., 2007).

#### 4.4.2 Cell wall

Both β‐expansins (Et_s2486‐1.7, Et_2486‐1.2, Et_s5792‐0.0, Et_s9915‐0.7) and xyloglucan endotransglucosylase/hydrolases (XTHs) (Et_s5823‐0.14, Et_s11804‐0.16) were upregulated in *Tsedey*. Scaffold2486 contains two tandem genes annotated by Blast2GO as expansin genes, in which only one was identified as an expansin by MapMan. With only two exons, the ambiguous gene looks incomplete, supporting the annotation of MapMan which annotates it as a gene of unknown function (Table 4). Under flooding conditions, rapid elongation of internodes and petioles is often observed as plants use an escape strategy to try to reach an environment with a better oxygen supply. Expansins and XTHs are involved in breaking down the cell walls to allow rapid expansion and growth (Li et al., 2002; Rose, Braam, Fry, & Nishitani, 2002; Sampedro & Cosgrove, 2005; Tsuchiya, Satoh, & Iwai, 2015) and have been shown to contribute to rapid coleoptile growth in rice (Miro & Ismail, 2013). One β‐expansin has been shown to induce the extension of maize coleoptiles (Cosgrove, Bedinger, & Durachko, 1997). Expansins have also been implicated in the formation of adventitious roots in the loblolly pine (*Pinus taeda*) (Hutchison, Singer, McInnis, Diaz‐Salaz, & Greenwood, 1999) and deepwater rice (Cho & Kende, 1997). The action of XTHs involves breaking linkages in the xyloglucan–cellulose network and then reforming them in a new position, allowing expansion without impairing overall wall integrity. XTH transcripts have been shown to increase in maize roots after 12 h of hypoxia due to flooding (Saab & Sachs, 1996).

Lysigenous aerenchyma result from the death of certain cells in the root cortex and are found in crops such as wheat, rice, barley, and maize (Shiono, Takahashi, Colmer, & Nakazono, 2008). Ethylene accumulation has been implicated as the primary signal in the formation of lysigenous aerenchyma (Shiono et al., 2008). This triggers a signal transduction pathway involving phosphoinositides and Ca^2+^ (Drew, He, & Morgan, 2000). Although no genes in this pathway have been detected to have differential expression under waterlogging, an XTH is upregulated (Table 4). An XTH has also been found to be associated with aerenchyma formation (Saab & Sachs, 1996). In maize, a mechanism for cortical cell‐specific programmed cell death has been proposed that includes the generation of ROS in combination with suppression of a metallothionein ROS scavenger. The consequent buildup of ROS leads to cell death and aerenchyma formation (Yamauchi, Rajhi, & Nakazono, 2011).

Addition of the regulated tef expansin sequences to the phylogenetic tree of Li et al. (2003) shows that the regulated tef sequence is of type β1, the most closely related to the Li sequences EXPβ1.10 which forms a clade with EXPβ1.7, EXPβ1.8, EXPβ1.9, and EXPβ1.11 (Fig. S11). Expression of β‐expansins has been correlated with internodal elongation in deepwater rice (Lee & Kende, 2001).

Peroxidases also affect cell wall extensibility. Correlations between the decrease in cell wall‐bound peroxidase activity and coleoptile elongation have been observed (Ismail, Ella, Vergara, & Mackill, 2009; Lee & Lin, 1995). Peroxidase activity has been found to be significantly higher in flooding‐sensitive genotypes of rice. Indeed, in the current study, a class III peroxidase, Et_s190.0.32, is also downregulated.

#### 4.4.3 Changes in carbohydrate metabolism

The MapMan category primary metabolism includes the metabolism of amino acids, nitrogen metabolism, sugar and its derivatives, and energy. Plant roots subjected to flood stress switch from aerobic respiration to fermentative metabolism, requiring soluble sugar, which is produced from carbohydrates stored as starches. Starch breakdown is the result of the action of the hydrolytic enzymes alpha amylase, β‐amylase, debranching enzyme, and alpha glucosidase (Guglielminetti, Yamaguchi, Perata, & Alpi, 1995). Several genes that function in the metabolism of sugar and its derivatives were upregulated under water stress. One is granule‐bound starch synthase (Et_s66‐0.13‐1 and Et_s6672‐0.28‐1), an enzyme that converts ADP‐glucose to amylose. This gene is more upregulated in *Tsedey* than in *Quncho* or *Alba* (Figure 5). Another upregulated gene is Et_2217‐0.39‐1, β‐amylase, which is active in the conversion of starch to maltose. Amylase activity has a positive correlation with both shoot and root elongation and with plant survival when comparing tolerant and non‐tolerant rice genotypes (Ismail et al., 2009). Upregulation of β‐amylase transcripts under waterlogging stress has also been observed in *Rumex* (van Veen et al., 2013).

The glucose‐6‐phosphate translocator precursor (Et_s2233‐0.29) is homologous to rice Os10g33920 and Os08g01410 and is also upregulated under waterlogging stress in tef. The expression of Glc6P/phosphate translocator 2 (GPT2) has been found to have a crucial role in the seedling response to exogenous sugars. Sugar transporters tend to be upregulated in shoots but downregulated in roots under flooding in model plant *Arabidopsis* (Dyson, Webster, & Johnson, 2014). Under the category minor carbohydrate metabolism, Et_s2617‐0.51 (aldo‐keto reductase family 4 member c9) was found to be downregulated. This is a member of a family of proteins with roles in metabolism and stress response.

#### 4.4.4 Metal handling

Excess water and oxygen depletion in the soil cause a sharp drop in the soil redox potential, creating reducing conditions and resulting in a buildup of Fe II and Mn II (Shabala, 2011). Et_s12869‐0.19 and Et_s13065‐0.28, annotated as chloroplast‐like ferric reduction oxidases, involved in the reduction of Fe III to Fe II, and maintaining iron homeostasis, were found to be upregulated. Other upregulated genes involving metals include Et_s1056‐0.47, a heavy metal‐associated domain‐containing protein often found in proteins involved in heavy metal transport or detoxification, Et_s7784‐0.11, annotated as isoform 1 of a magnesium proton exchanger, and Et_s786‐1.34, a pap‐specific phosphatase, which has the GO molecular function of magnesium ion binding and the MapMan function of degradation.

#### 4.4.5 Secondary metabolism (response to reactive oxygen species)

Both the pathway for the synthesis of phenylpropanoids, specifically lignin, and the pathway for the synthesis of carotenoids have enzymes that are upregulated under flooding stress. In the volatile carotenoid pathway of MapMan, isoprenoids.carotenoids.phytoene synthase, Et_s517‐0.16‐1 is upregulated 2.75 times. Its function is to convert GGPP into phytoene. In the volatile phenyl propanoid pathway (secondary metabolism phenylpropanoid lignan biosynthesis), two genes are upregulated. The first is Et_s20148‐0.9‐1 [cinnamoyl CoA reductase 1 (CCR1)], which is involved in lignin biosynthesis and probably involved in the formation of lignin in defense responses in rice (Kawasaki et al., 2006).

Lignin, a component of the plant cell wall, has functions associated with mechanical support, water transport, and defense against pests. Both biotic and abiotic stresses are known to invoke changes in lignin content and composition in plants (Moura, Bonine, Viana, Dornelas, & Mazzafera, 2010). The development barrier to ROL is often associated with suberization and sometimes also lignification of the outer part of the roots (Abiko et al., 2012).

The second gene upregulated in this pathway is Et_c8513699‐0.0‐1 (isoflavone‐7‐O‐methyltransferase), implicated in the biosynthesis of phenylpropanoids. Alfalfa transformants overexpressing isoflavone O‐methyltransferase have increased induction of phenylpropanoid/isoflavonoid pathway gene transcripts after infection with *P. medicaginis* and also have increased resistance (He & Dixon, 2000).

Another upregulated antioxidant is Et_s4037‐1.43, a GDP‐1‐galactose phosphorylase 2‐like protein which catalyzes a step in the biosynthetic pathway of vitamin C, an important antioxidant. Recently, oxidative stress was suggested to be a major tolerance factor in submergence‐tolerant *Brachypodium distachyon*, and ascorbate oxidases and ascorbate peroxides were among the upregulated antioxidants (Rivera‐Contreras et al., 2016; Ushimaru et al., 1997; Zhang et al., 2015).

#### 4.4.6 Stress tolerance

Proteins known to be involved in the abiotic stress response had mixed changes in expression. Two salt tolerance‐like proteins, Et_s7183‐0.17 and Et_s6551‐0.36, were upregulated as was Et_s304‐1.36, a late embryogenesis abundant protein (LEA5‐like) that was annotated by Blast2GO but not by MapMan. Salt stress and waterlogging stress often occur together on irrigated land as waterlogged soils prevent leaching of the salts brought by the irrigation water. Adequate drainage is effective in reducing both problems. However, as tef is not usually irrigated, the likelihood that breeding has selected for both traits is small.

Downregulated genes under waterlogging were Et_s346‐4.13 and Et_s3572‐0.37, annotated as bifunctional nuclease 2‐like isoform ×1 and annotated by MapMan as being involved in touch/wounding stress. Expression measurements indicated that the bifunctional nuclease 2‐like gene is downregulated more in *Tsedey* than in *Alba* or *Quncho* (Figure 5). A chaperone protein 1‐like (Et_s11116‐0.0) involved in heat stress was also downregulated.

#### 4.4.7 RNA regulation

Three proteins are categorized into MapMan category 27, RNA regulation. The first is Et_s3159‐0.24, a ribonuclease s‐like protein precursor, downregulated 3.5 times. This sequence was added to the phylogenetic tree of ribonuclease sequences produced by MacIntosh, Hillwig, Meyer, & Flagel (2010) in Fig. S12, which shows that the tef ribonuclease groups with the class 1 ribonucleases. In general, class I ribonucleases show tissue specificity and are associated with stress regulation. Interestingly, it appears in a monocot‐specific class I S‐like clade, which includes proteins that have lost their ribonuclease activity due to the loss of two catalytic histidines. Although they are not active ribonucleases, they are expressed and the rice homolog is expressed under drought conditions (Salekdeh, Siopongco, Wade, Ghareyazie, & Bennett, 2002) indicating a change in function. Indeed, both of these histidines have undergone substitution in the tef homolog.

In addition, a TUBBY‐like f‐box protein type 7, Et_s2666‐0.25 is downregulated approximately by a factor of 3. F‐box proteins are characterized by a conserved F‐box domain, which has a length of about 40 amino acids. A subfamily of these proteins contains a TUBBY domain. The *Arabidopsis* TUBBY‐like protein type 7 may play a role in regulating phytohormone signaling and appears to be regulated by a homeobox transcription factor, KN1. In addition, it was found to be downregulated in *Arabidopsis* under conditions of auxin induction (naphthylacetic acid‐treated roots) and an excess of potassium (Lai et al., 2004).

#### 4.4.8 Signaling and transport

Aquaporins form a family of membrane proteins that allow control of water flow through a membrane either by changing the abundance of these proteins in the membrane or by changing the rate of flow through the pores. Aquaporin activity may be key in influencing water transport through waterlogged roots (Bramley, Turner, Tyerman, & Turner, 2007) and may be a critical part of determining a plant's isohydric characteristics, that is, how much they maintain the same water potential during variations in water availability. In the current work, two aquaporins of the TIP4 subtype, Et_s819‐0.0 (TIP4‐2) and Et_s4179‐0.34 (TIP4‐1), were downregulated. Downregulation of the PIP and TIP aquaporin subtypes under waterlogging stress has been found in *Arabidopsis* (Liu et al., 2005). In sorghum, waterlogging‐tolerant genotypes showed differential regulation of PIP2‐6, PIP2‐7, TIP2‐2, TIP4‐4, and TIP5‐1. However, SbTIP4‐4 was downregulated in the sensitive genotypes (Kadam et al., 2017). TIP2 regulates the response to abiotic stresses (salt and drought) in bread wheat (*Triticum aestivum*) (Kayum et al., 2017) and tomato (*Solanum lycopersicum*) (Sade et al., 2009). In addition to the aquaporins, some proteins involved in metal and nitrate transport (Et_s7784‐0.11, Et_s6198‐0.9, Et_s594‐1.9) were upregulated.

#### 4.4.9 Not found

Surprisingly, none of the stress‐induced hormones in the ABA, GA, or JA pathways had detectable changes in expression after 9 days of waterlogging stress. Ethylene has been shown to play a role in both the induction of the GA pathway (Voesenek et al., 2003) and aerenchyma formation in maize and rice (Nishiuchi et al., 2012). Gene regulation in *Arabidopsis* shoots under flooding was also affected in a mutant with an ethylene signaling mutation. In addition, genes associated with ABA biosynthesis were upregulated in shoots and downregulated in roots, indicating a role for ethylene in the response. An ABA signaling mutation, abi4‐1, affected the expression of several systemic responsive genes, suggesting that ABA biosynthesis may be part of the systemic response to flood response (Hsu, Chou, Peng, Chou, & Shih, 2011).

## 5 CONCLUSIONS

We report here on the morphology, growth, physiology, and differential gene expression in tef under conditions of long‐term water stress starting at the tillering stage and early in development. Although tef has been reported to be waterlogging tolerant, this is the first quantification of changes to gene expression, morphology, and physiology under waterlogging. Three tef genotypes, two improved varieties and one landrace, were investigated and differences in waterlogging tolerance among the three genotypes were found. The improved variety *Tsedey* was found to have increased root and shoot biomass under waterlogging conditions when waterlogged in early development. It formed more adventitious roots and aerenchyma than the other two genotypes and is a promising candidate for further study. Differences in plant growth characteristics peaked at 9–11 days after the onset of stress for early waterlogging. Differential expression of 19‐day‐old seedlings waterlogged at the tillering stage shows changes in the cell wall, carbohydrate metabolism, and upregulation of genes involved in the response to ROS. In addition, genes affecting transport and lignification were affected. The identification of lines more tolerant to waterlogging conditions will aid the cultivation of cereal crops in poorly drained soils as tolerant waterlogging lines will be introgressed to elite tef genotypes, and the relevant genetic loci may be introduced to other cultivated crops. As this study shows that there is significant genetic diversity in tef genotypes with respect to waterlogging tolerance, a large‐scale screening is underway to identify and quantify the performance of 500 tef genotypes under waterlogging stress.

## AUTHOR CONTRIBUTIONS

G.C., A.W., and Z.T. conceived experiments; G.C., M.S., C.R., A.W., R.B., S.B., S.P.W., and Z.T. performed experiments; G.C., M.S., and C.R. analyzed the data; G.C. wrote the manuscript; all authors contributed to manuscript revision; S.C. and K.A. contributed to the original research plans; Z.T. conceived the original research plans.
