# Supplementary material for: Waterlogging affects plant morphology and the expression of key genes in tef (Eragrostis tef)
Source: Plant Direct. 2018 Apr 25;2(4):e00056. doi: 10.1002/pld3.56 (PMC6508588; doi:10.1002/pld3.56)
Supplement: Supplementary file 1 [file PLD3-2-e00056-s001.pdf]

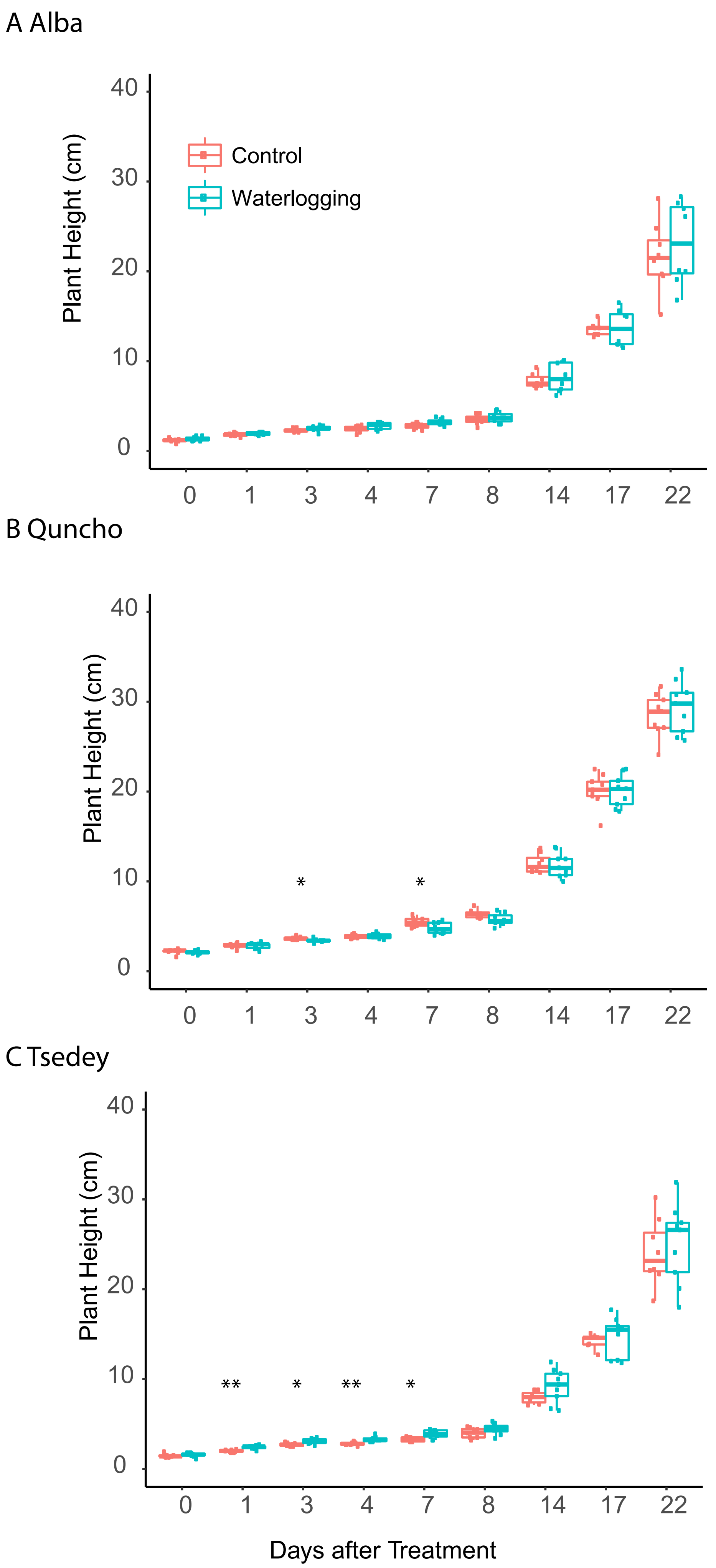

Figure S1. Growth of four-day old seedlings of two tef genotypes, Quncho and Tsedey, subjected to waterlogging and normal watering for 22 days. Shoot dry weight (A), root dry weight (B), Shoot length (C), Root length (D) and the number of leaves were measured from 0 to 24 days after treatment. Means marked with an asterisk are significantly different from each other (Mann-Whitney: * $p <= 0.05$).

16 days after waterlogging

Waterlogging control

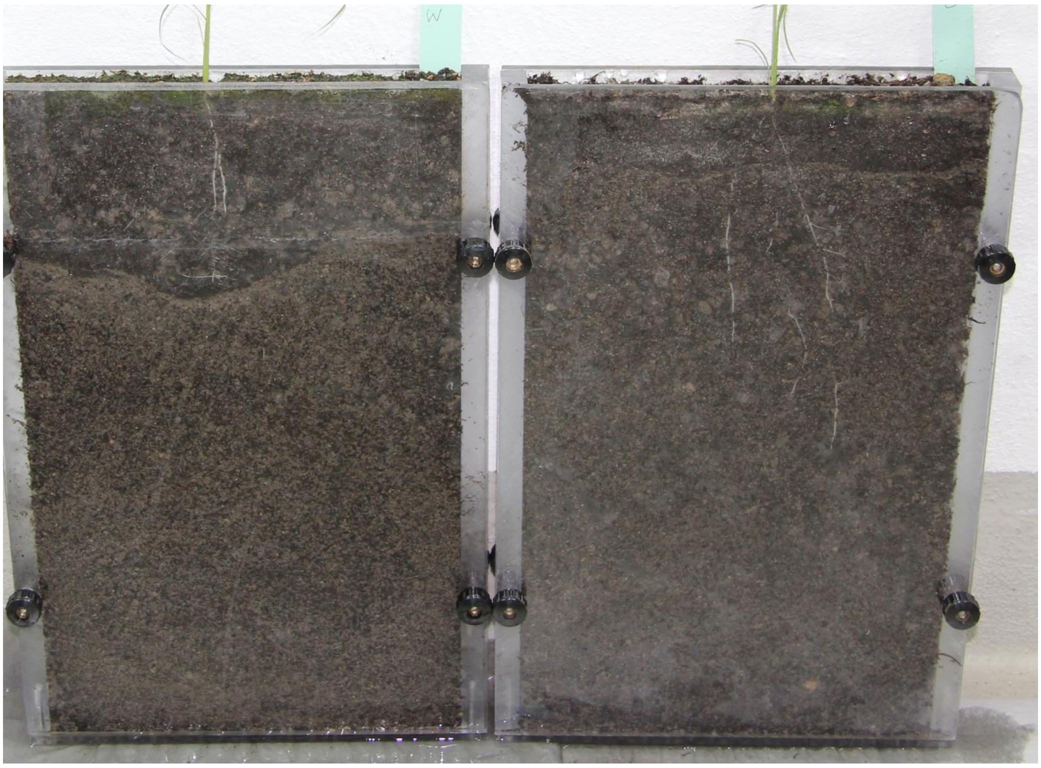

21 days after waterlogging

Waterlogging control

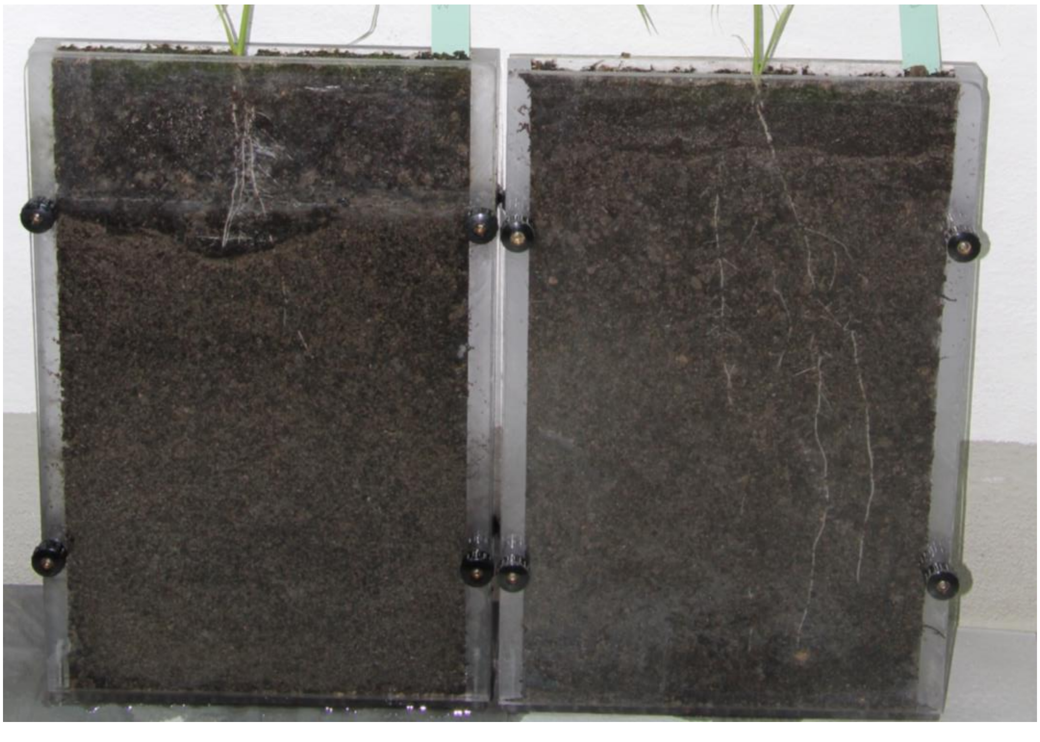

23 days after waterlogging

Waterlogging   control

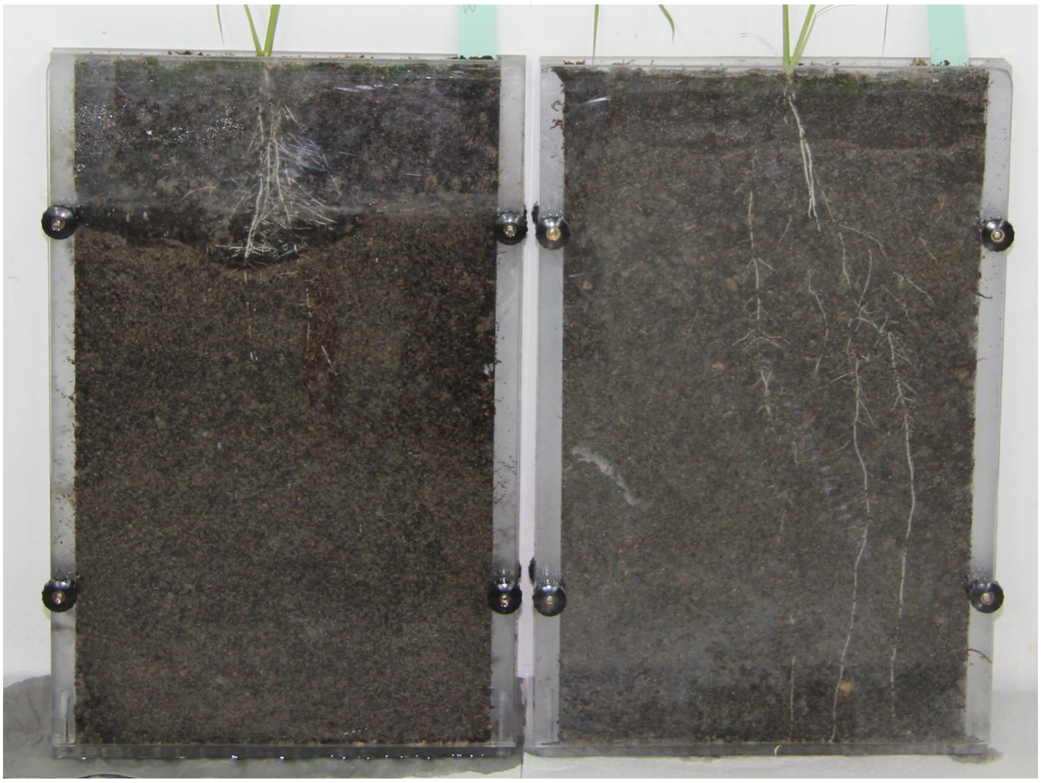

Figure S2. Visualization of *Tsedey* roots using a rhyzotron. Visualization of waterlogged (left) and control (right) *Tsedey* roots at from 16, 21 and 23 days after waterlogging 4 day-old seedlings.

1 day after waterlogging

Wheat waterlogging

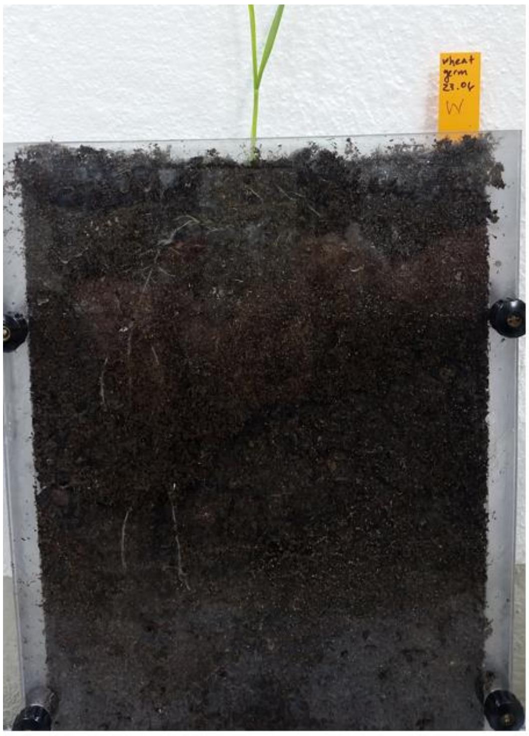

Wheat control

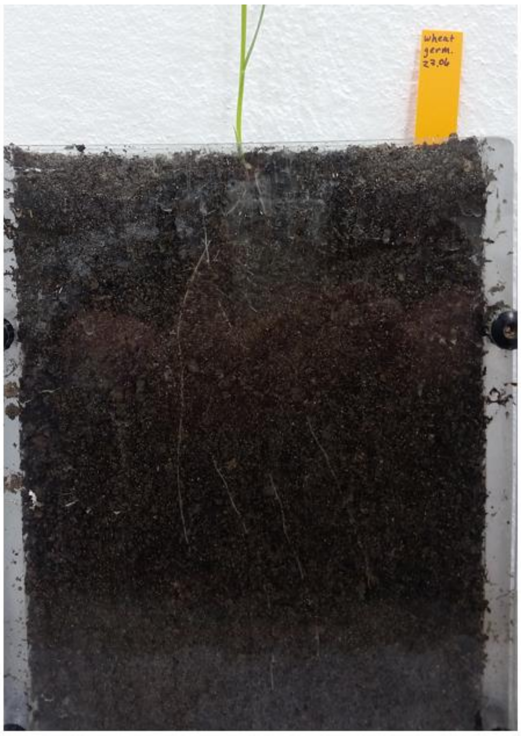

3 days after waterlogging

Wheat waterlogging Wheat control

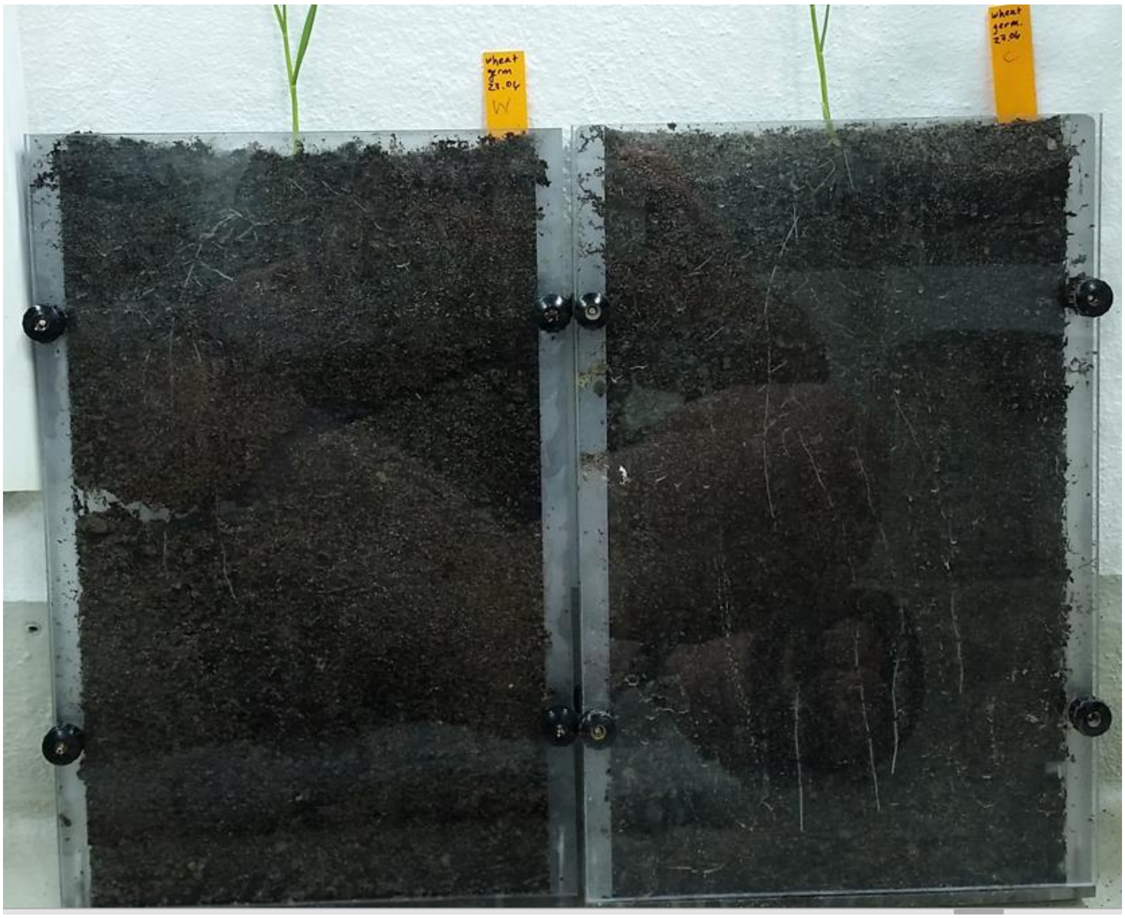

7 days after waterlogging

Wheat waterlogging

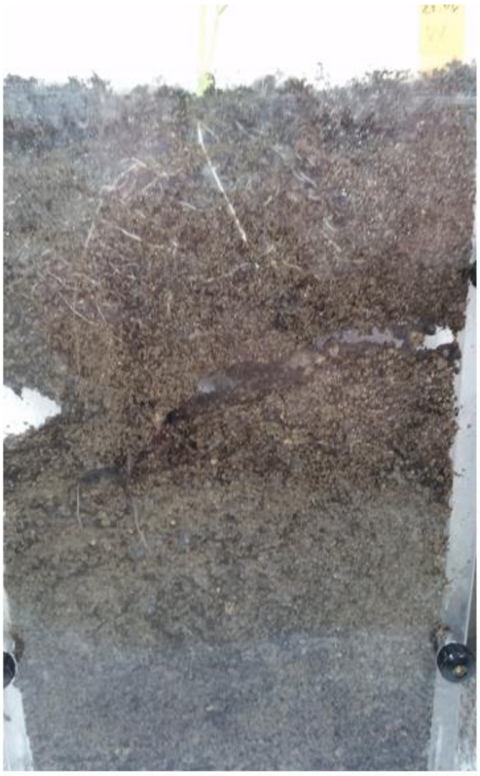

Wheat control

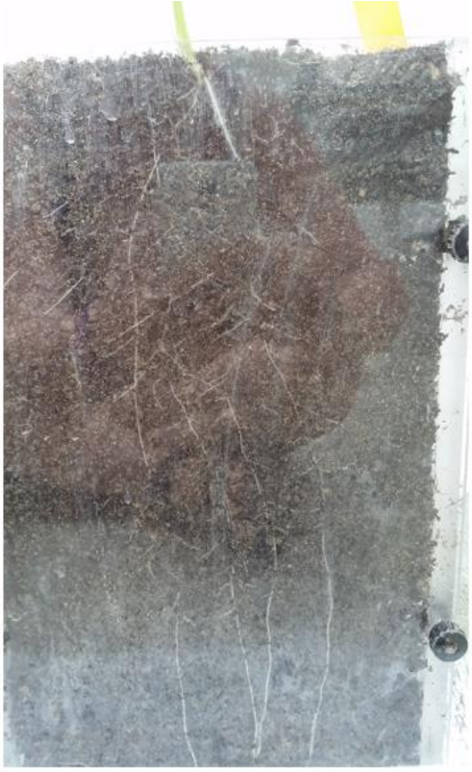

16 days after waterlogging

Wheat waterlogging Wheat control

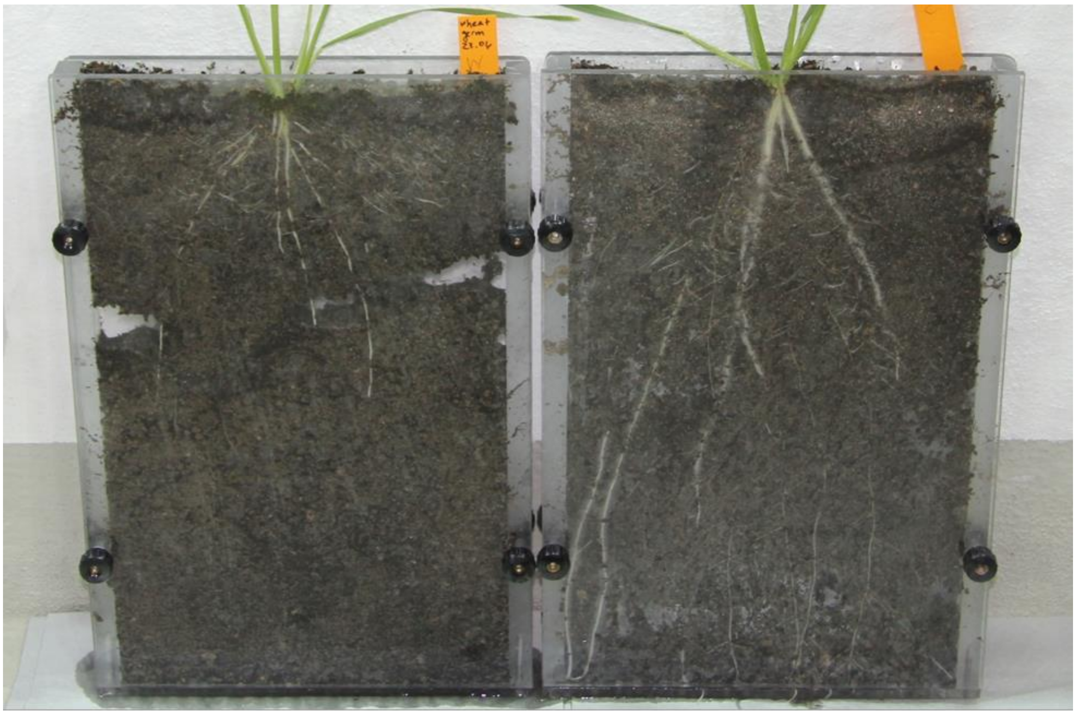

25 days after waterlogging

Wheat waterlogging Wheat control

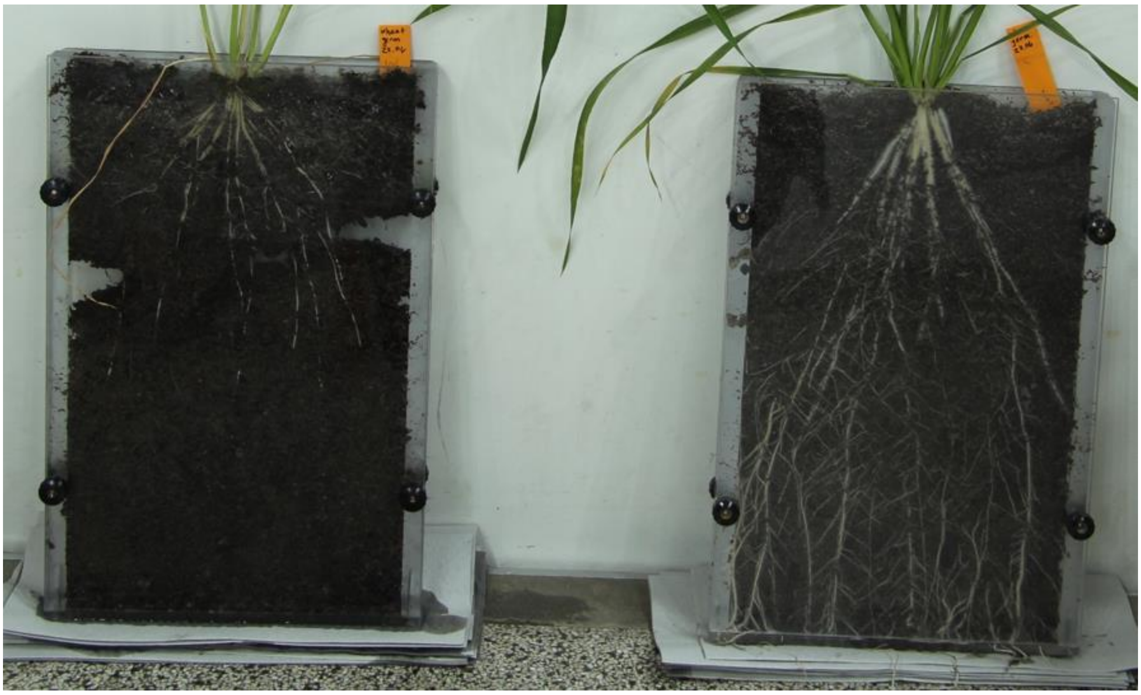

32 days after waterlogging

Wheat waterlogging Wheat control

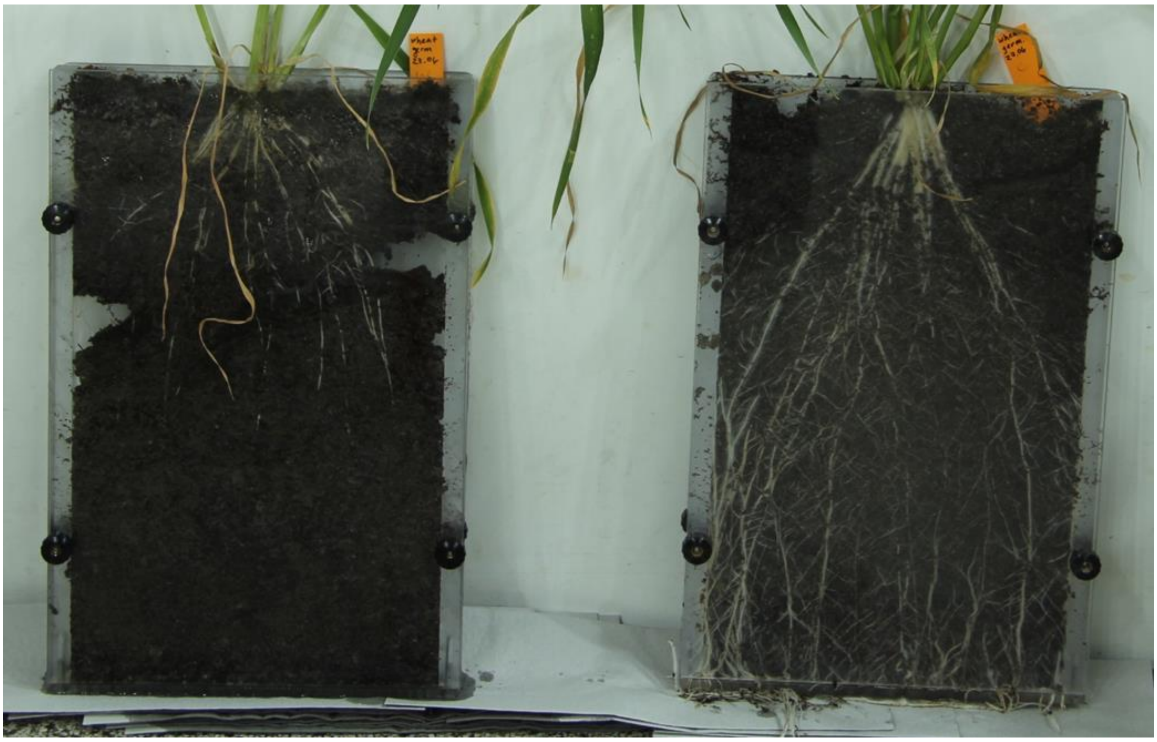

Figure S3. Visualization of Wheat roots using a rhyzotron. Visualization of wheat roots at 1, 3, 7, 8, 16, 25 and 32 days after waterlogging 7 day-old seedlings.

1 day after waterlogging

Maize waterlogging

Maize control

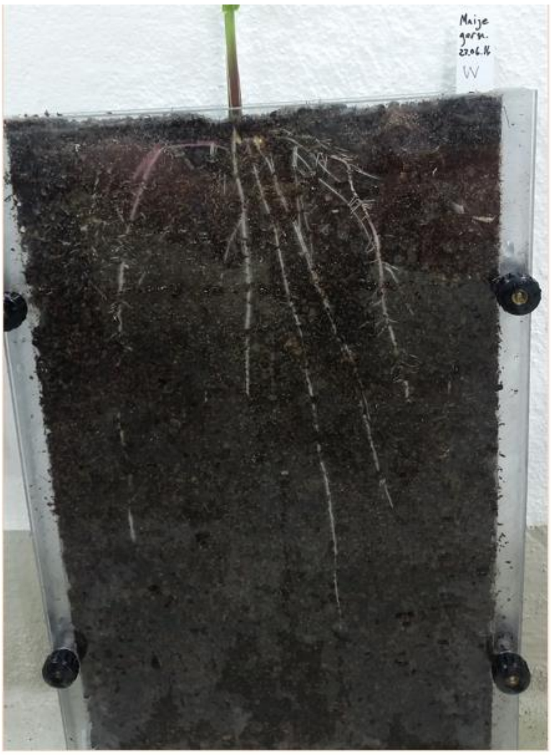

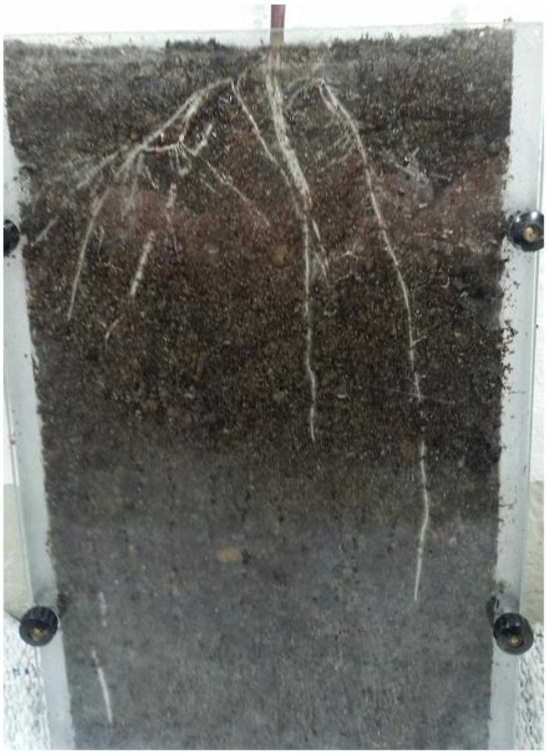

3 days after waterlogging

Maize waterlogging

Maize control

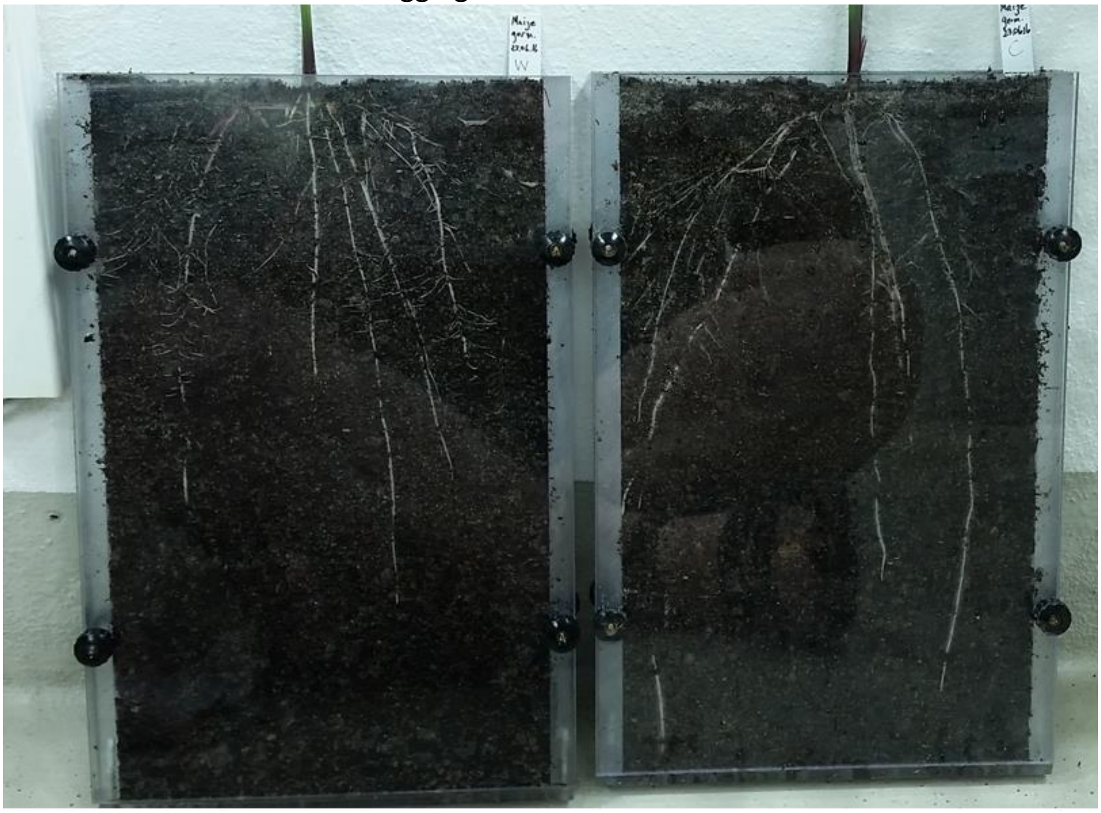

7 days after waterlogging

Maize waterlogging

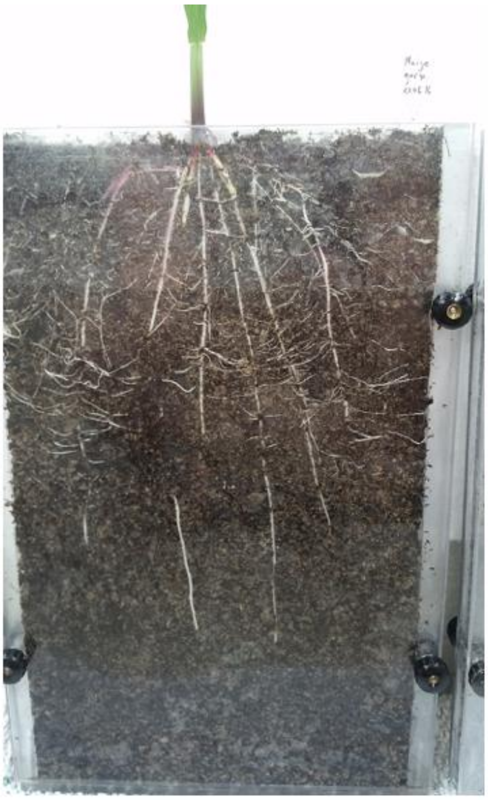

Maize control

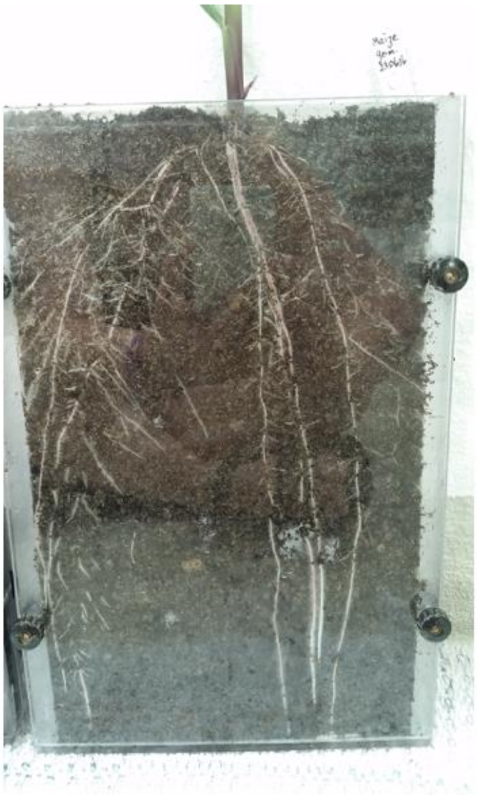

10 days after waterlogging

Maize waterlogging

Maize control

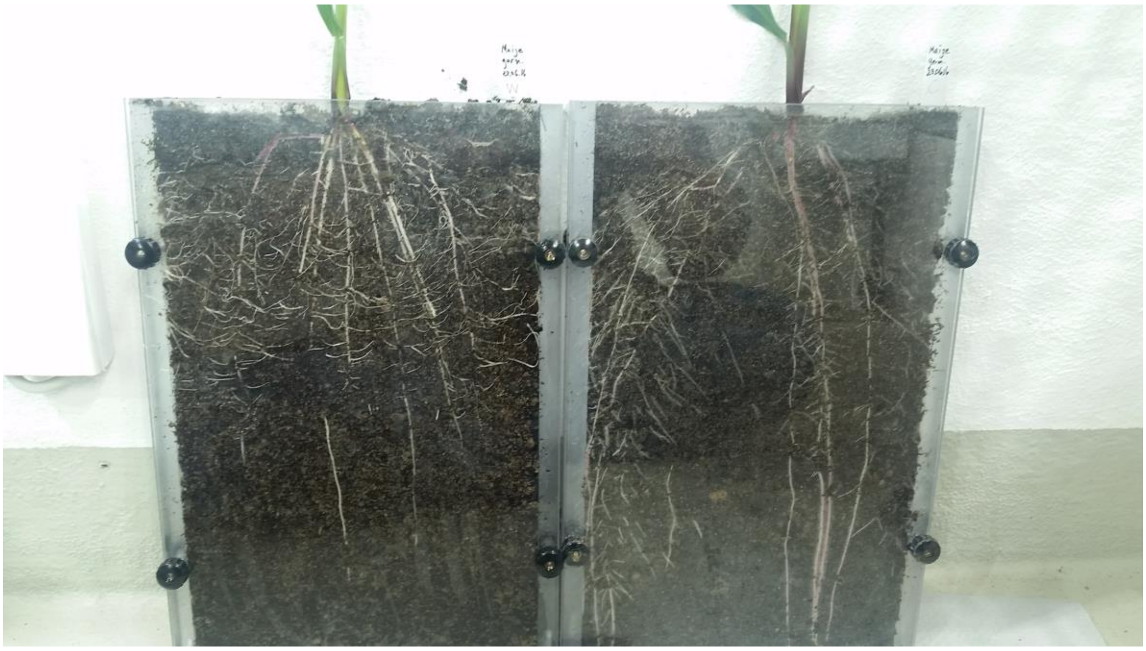

18 days after waterlogging

Maize waterlogging Maize control

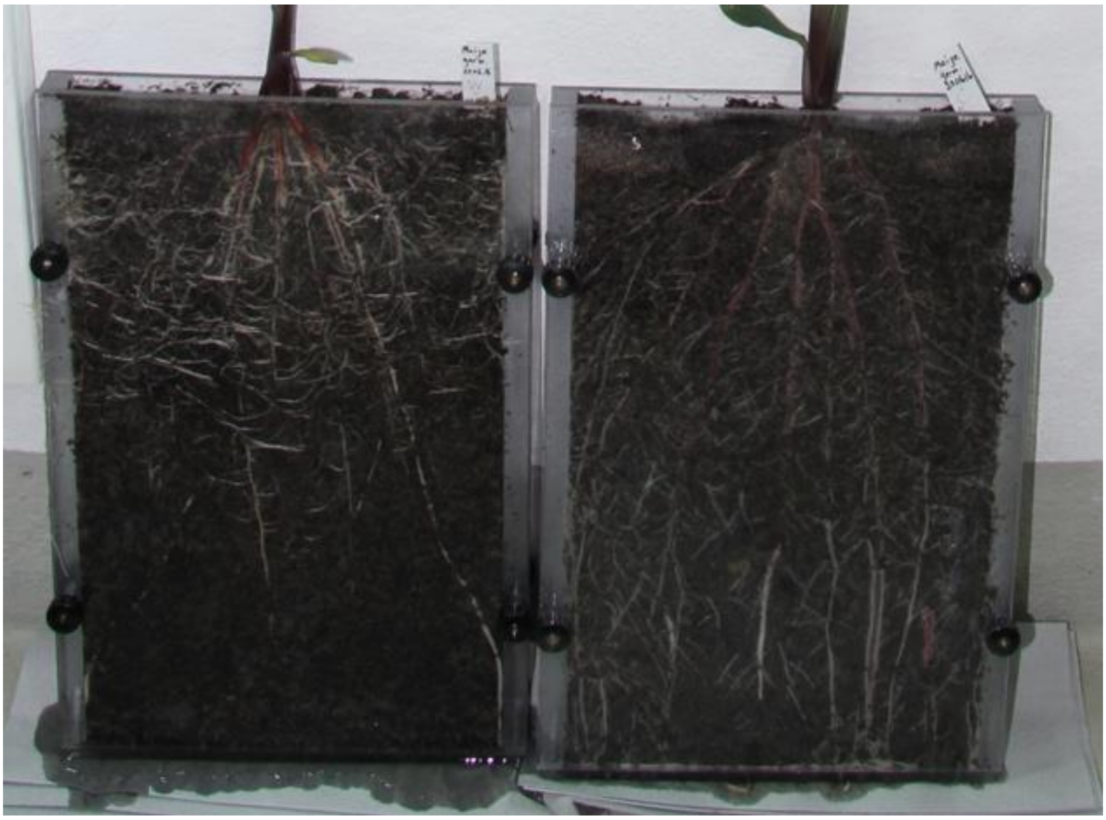

25 days after waterlogging

Maize waterlogging Maize control

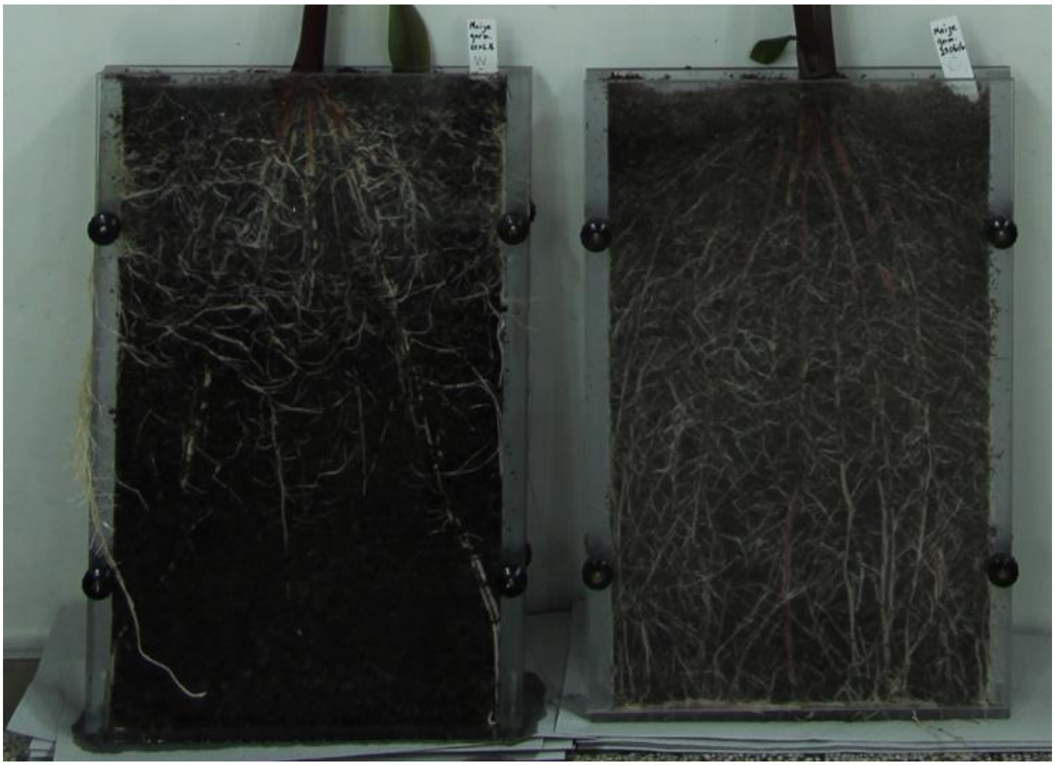

Figure S4. Visualization of Maize roots using a rhyzotron. Visualization of maize roots at 1, 3, 7, 10, 18, 21, 25 and 32 days after waterlogging 7 day-old seedlings.

Alba control					
A2_oben_1_10x 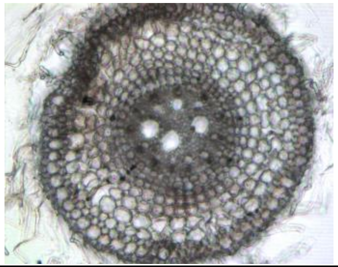	A2_oben_2_10x 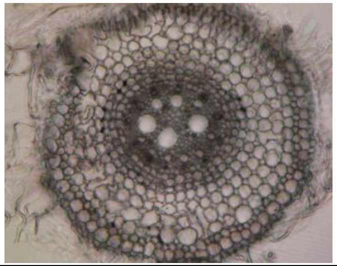	A8_oben_1_10x 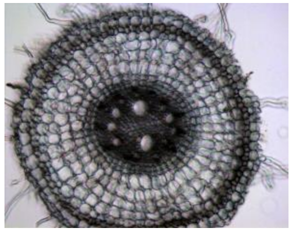			
A8_mitte_1_10x 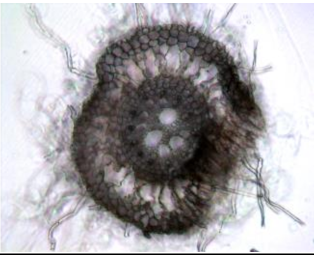	A8_mitte_2_10x 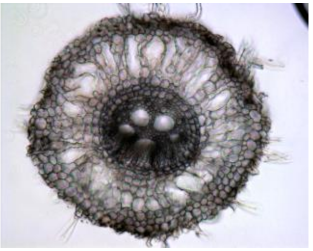	A8_mitte_3_10x 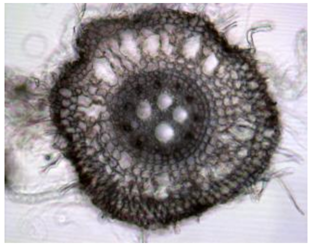			
Unten A1_unten_1_10x. 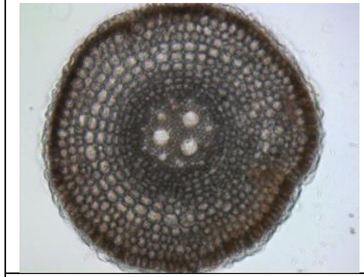	A1_unten_2_10x 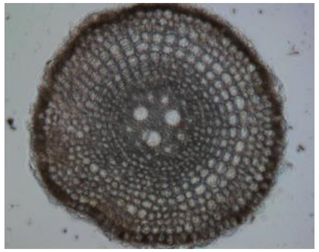	A1_unten_2_10x 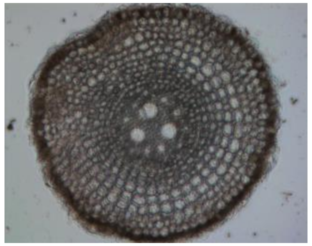	A2_unten_1_10x 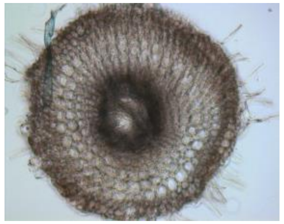	A2_unten_2_10x 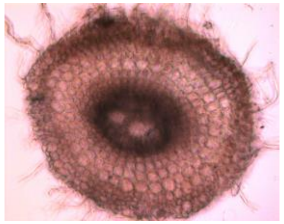	A8_unten_1_10x 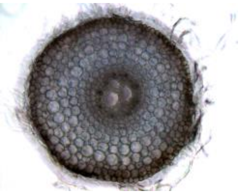
	A8_unten_2_10x 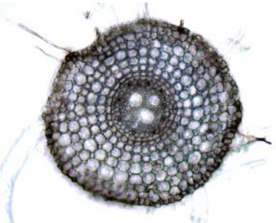				

Alba Waterlogging					
A13_oben_1_10x_200um<br>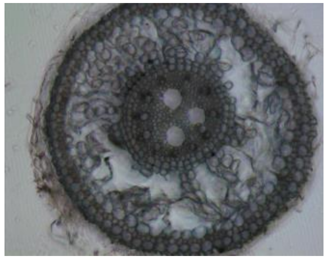	A13_oben_1_10x<br>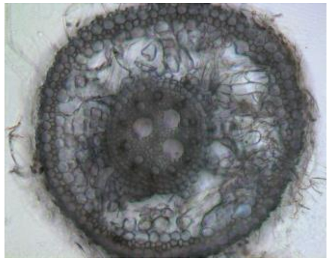	A13_oben_1_10x<br>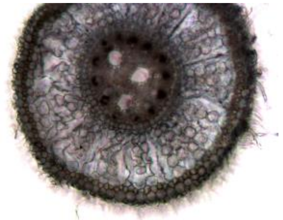	A13_oben_2_10x<br>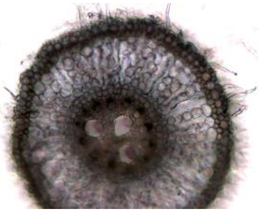	A13_oben_3_10x<br>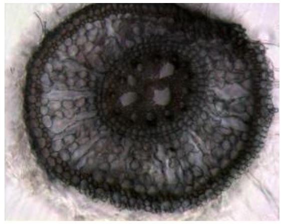	A16_oben_1_10x<br>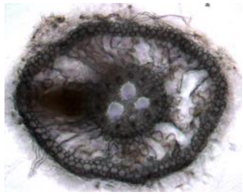
	A17_oben_1_10x<br>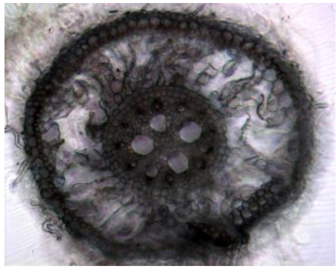	A17_oben_2_10x<br>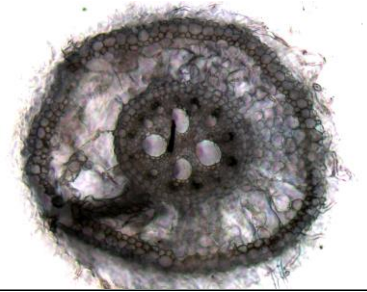			
Mitte A11_mitte_1_10x<br>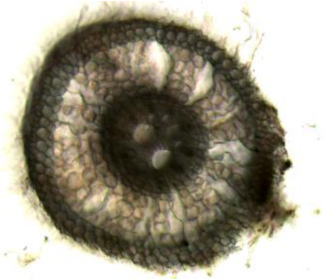	A11_mitte_2_10x<br>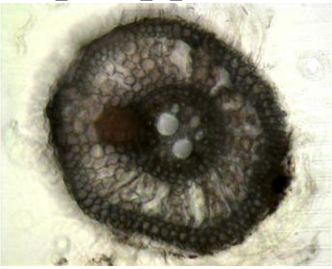	A11_mitte_3_10x<br>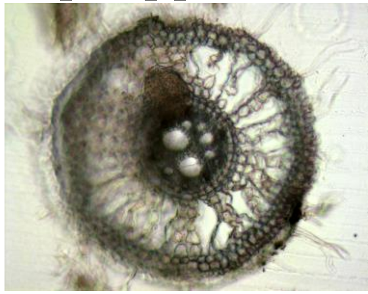	A11_mitte_4_10x<br>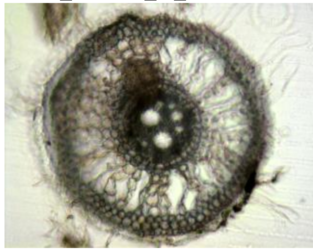	A11_mitte_5_10x<br>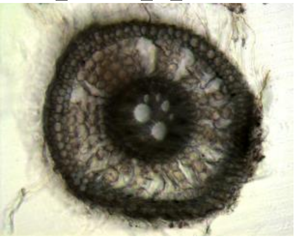	A17_mitte_1_10x<br>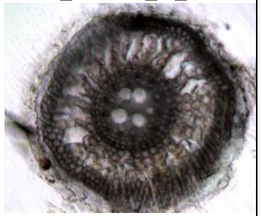
	A17_mitte_2_10x<br>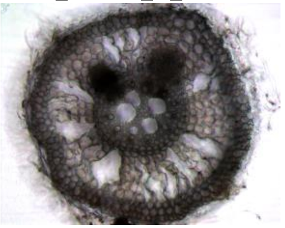				
Unten A13_unten_1_10x	A13_unten_2_10x	A11_unten_1_10x	A11_unten_2_10x	A11_unten_3_10x	A11_unten_4_10

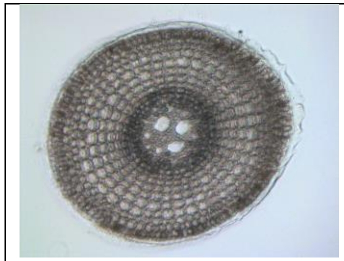	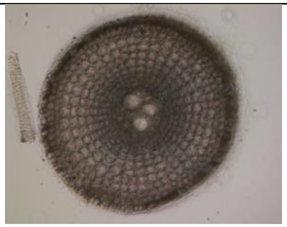	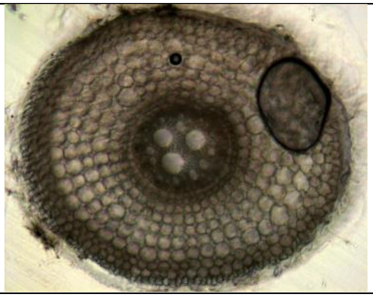	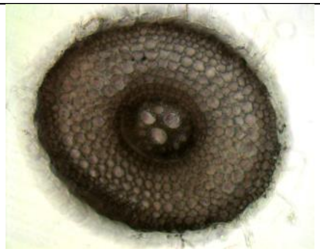	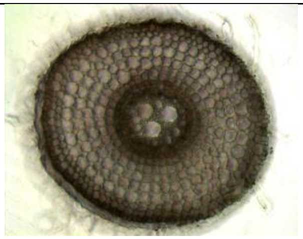	x 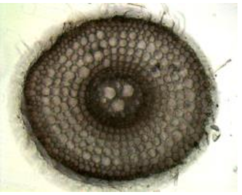
	A17_unten_1_10x 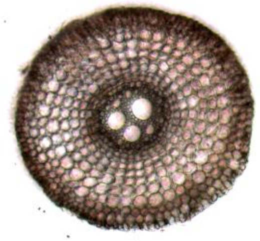				

Quncho control					
Q1_oben_1_10x 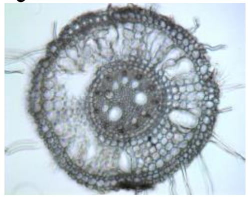	Q2_oben_1_10x 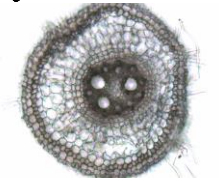	Q2_oben_1_10x 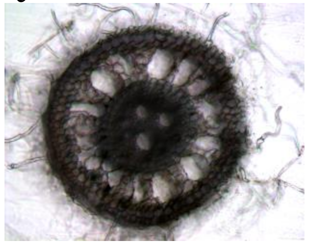	Q2_oben_2_10x 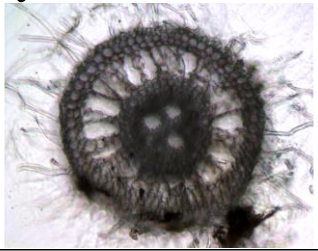		
Mitte Q1_mitte_1_10x 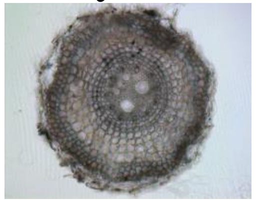	Q2_mitte_1_10x 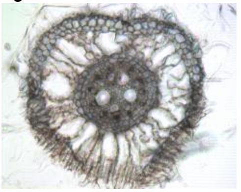	Q9_mitte_1_10x 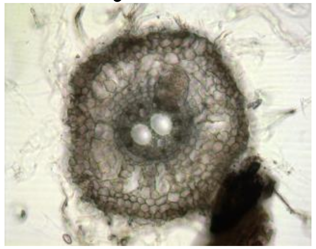	Q9_mitte_2_10x 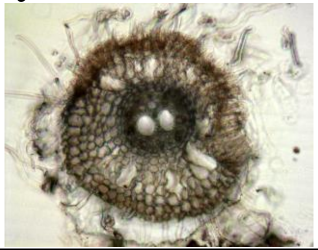	Q9_mitte_3_10x 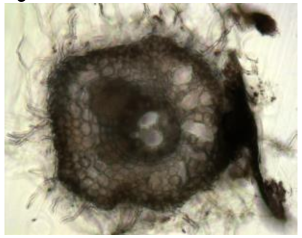	Q9_mitte_4_10x 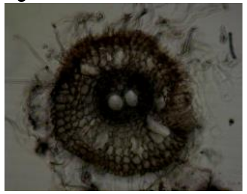
	Q9_mitte_5_10x 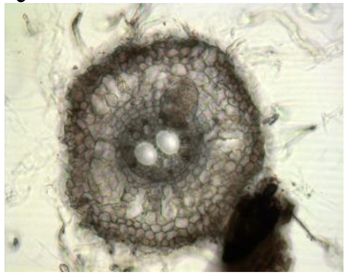				
Unten Q1_unten_1_10x 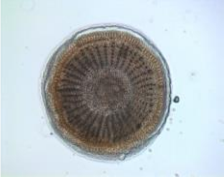	Q2_unten_1_10x_150u m 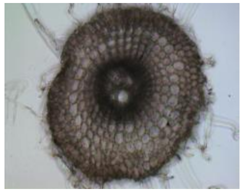	Q2_unten_1_10x 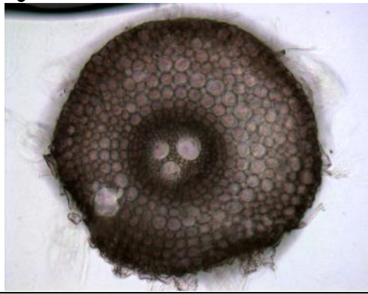	Q2_unten_2_10x 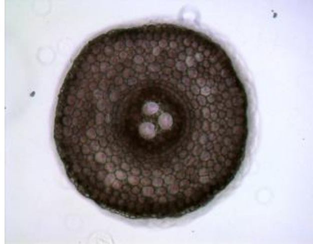	Q2_unten_3_10x 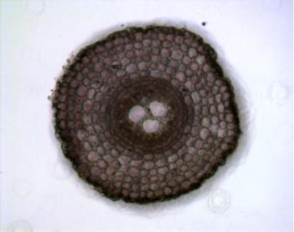	

Quncho Waterlogging					
Oben Q12_oben_1_10x 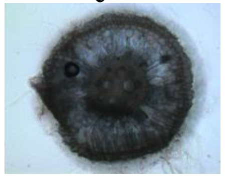	Q14_oben_1_10x 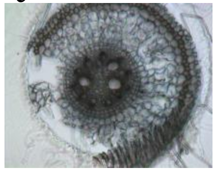	Q12_oben_1_10x 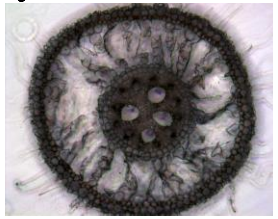	Q12_oben_2_10x 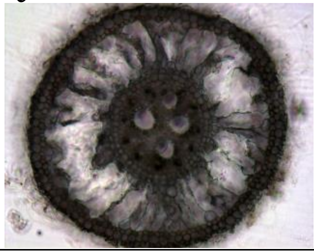	Q12_oben_3_10x 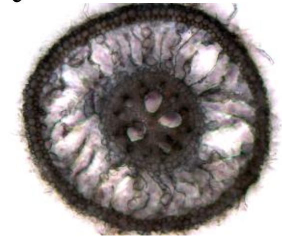	
Mitte Q12_mitte_1_10x 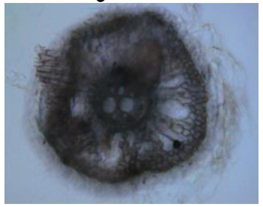	Q12_mitte_1_10x 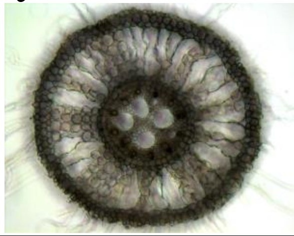	Q12_mitte_2_10x 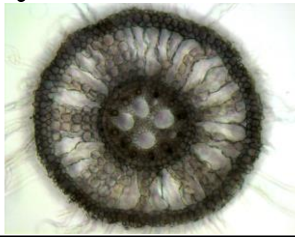	Q12_mitte_3_10x 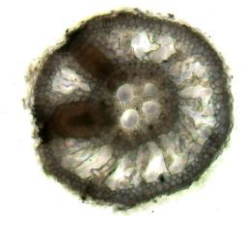	Q13_mitte_1_10x 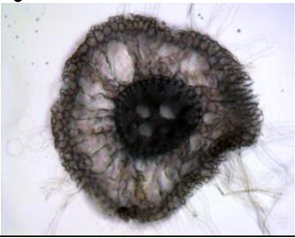	Q13_mitte_2_10x 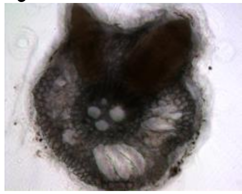
Unten Q12_unten_1_10x 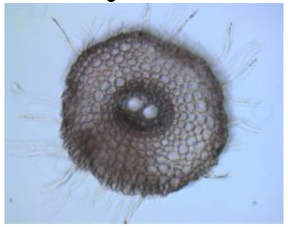	Q12_unten_2_10x 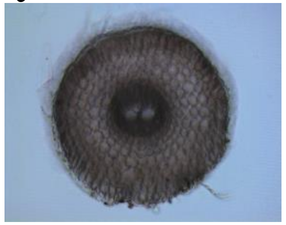	Q11_unten_1_10x 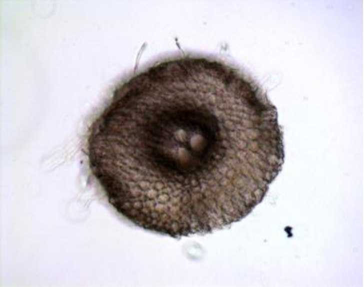	Q12_unten_1_10x 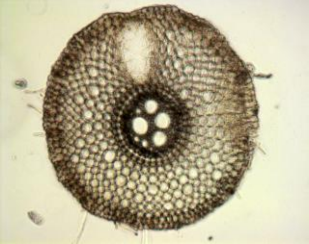		

Tsedey Control					
T5_oben_1_10x 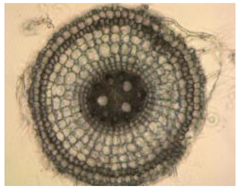	T5_oben_2_10x 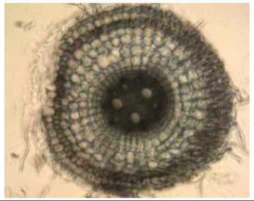	T5_oben_3_10x 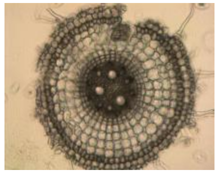	T9_oben_1_10x 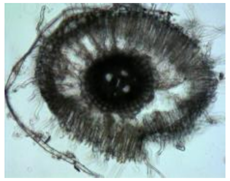		
Mitte T1_mitte_1_10x_150um 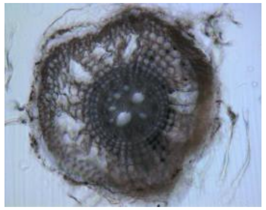	T1_mitte_2_10x_150um 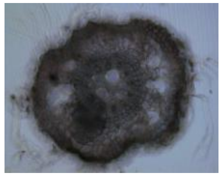	T5_mitte_2_10x 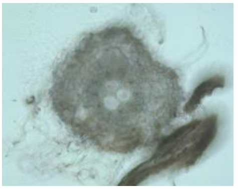	T5_mitte_3_10x 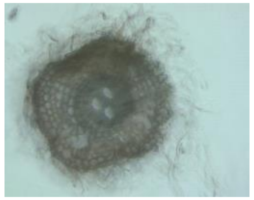	T5_mitte_4_10x 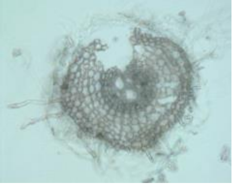	T5_mitte_10x 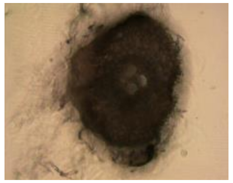
T9_mitte_1_10x 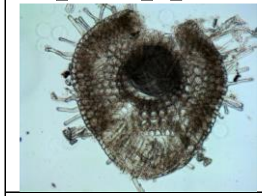					
Unten T1_unten_1_10x_150um 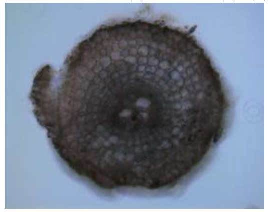	T5_unten_1_10x 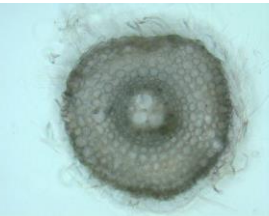	T5_unten_3_10x 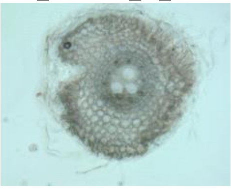	T5_unten_4_10x 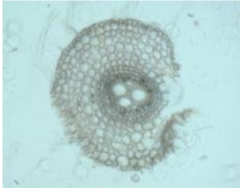	T9_unten_1_10x 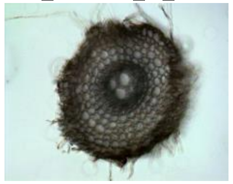	T9_unten_2_10x 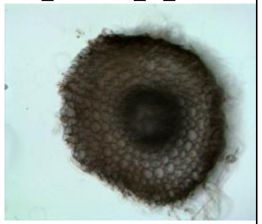

Tsedey Waterlogging					
T13_2_oben_1_10x 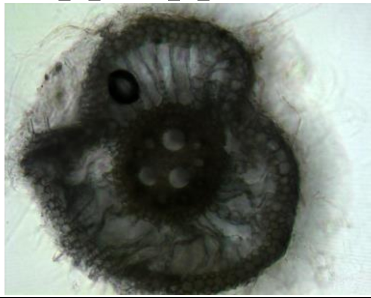	T13_2_oben_2_10x 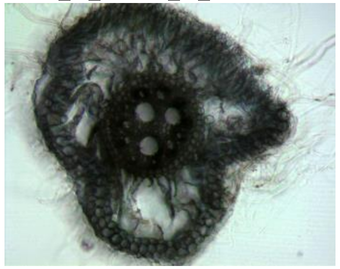	T13_oben_1_10x 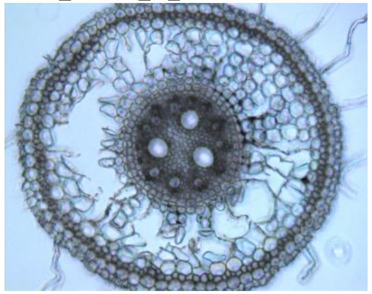	T13_oben_2_10x_150 um 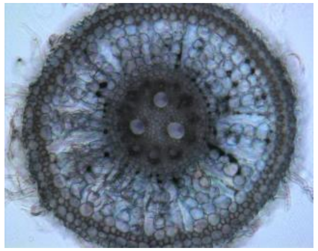	T13_oben_3_10x_150 um 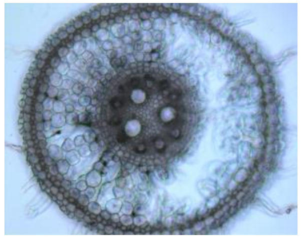	T14_oben_1_10x 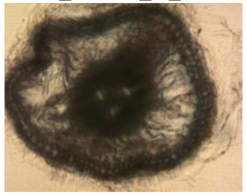
	T16_oben_1_10x 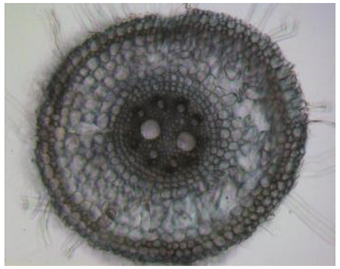	T16_oben_2_10x 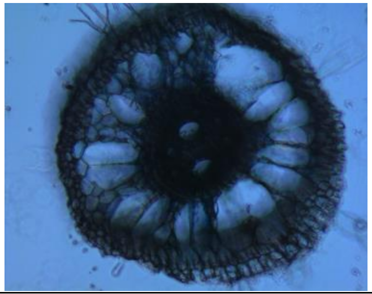			
T13_2_mitte_1_10x 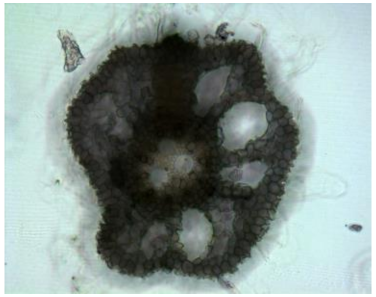	T13_mitte_1_10x_150u m 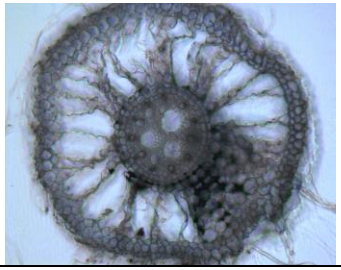	T13_mitte_2_10x_150um 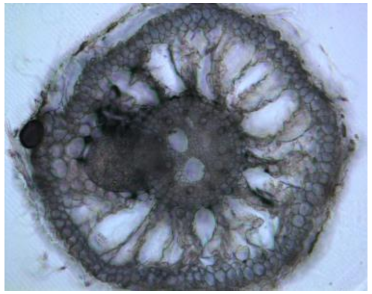			
T13_unten_1_10x_150um 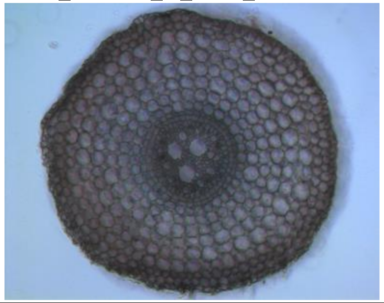	T14_unten_1_10x 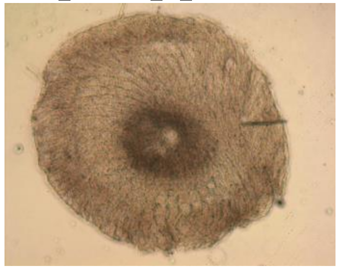	T16_unten_1_10x 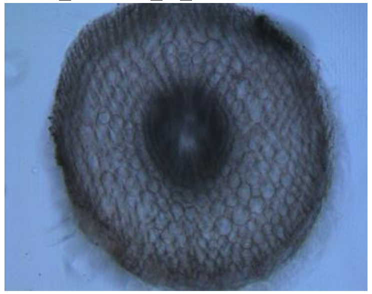	T16_unten_2_10x 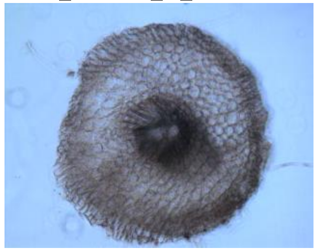		

Figure S5. Complete set of cross sections taken from 28-day old plants. Cross sections of 19 day-old *Alba*, *Tsedey* and *Quncho* subjected to 9 days of waterlogging stress. All cross sections photographed are included for visualization of the variability.

Days after treatment	tip		middle		top	
	control	waterlog	control	waterlog	control	waterlog
2<br>20x	Quncho_58_tip_1502_150um_20x<br>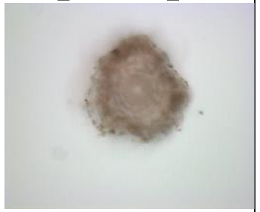	Quncho_8_tip_1502_150um_20x<br>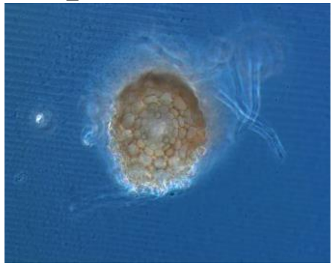			Quncho_58_up_1502_150um_10x<br>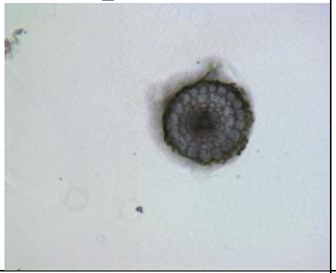	Quncho_8_up_1502_150um_2<br>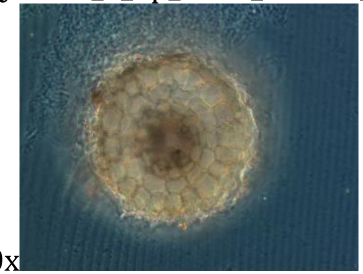<br>0x
4<br>20x	Quncho_66_2nd tip_1702_150um_20x<br>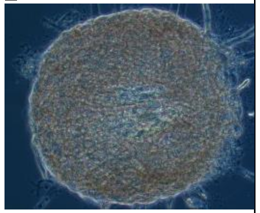	Quncho_29_2nd tip_1702_150um_20x.tif<br>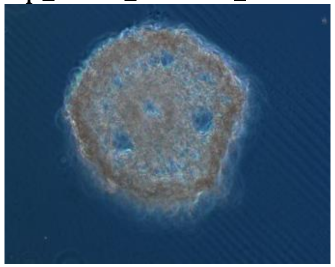			Quncho_66_2nd up_1702_150um_20x.tif<br>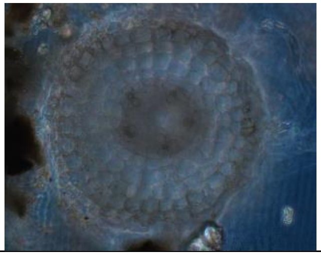	Quncho_29_2nd up_1702_150um_20x.tif<br>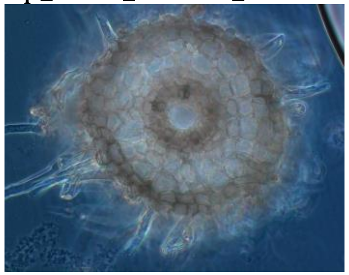
7<br>20x		Quncho_32_2nd tip_200um_20x<br>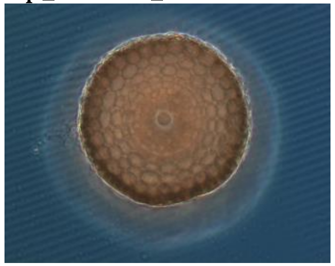	Quncho_49_2nd middle_150um_20x.tif<br>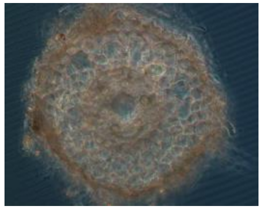	Quncho_32_2nd middle_150um_20x.tif<br>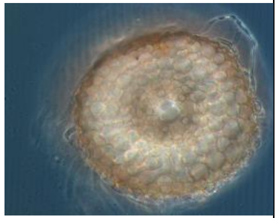	Quncho_49_2nd up_150um_20x<br>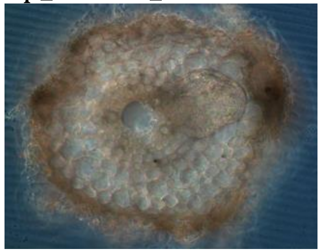	Quncho_32_2nd up_150um_20x<br>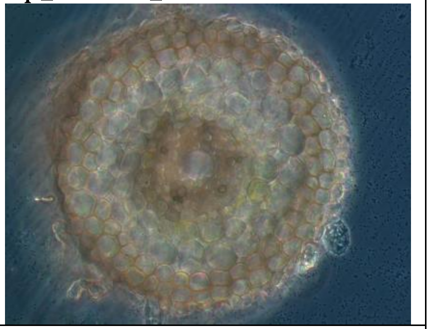
9<br>20x	Quncho_45_2nd tip_150um_20x.tif		Quncho_45_2nd middle_100um_20x.tif	Quncho_19_2nd middle_150um_20x		

	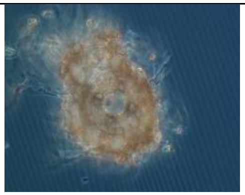		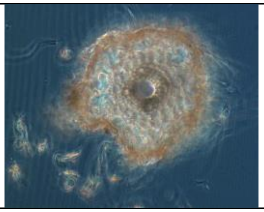	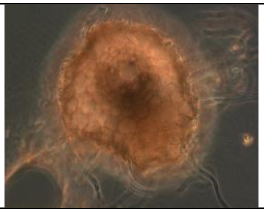		
16	Quncho_42_ top_150um_10x_ 1 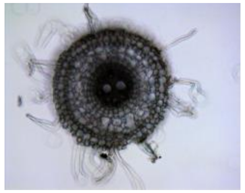	Quncho_20_ tip_150um_20x_2 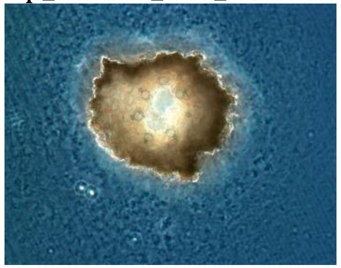	Quncho_42_ middle_150um_10 x_2 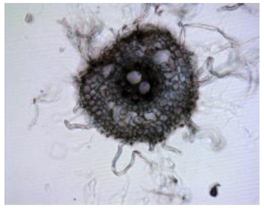	Quncho_20_ middle_150um_20 x_2 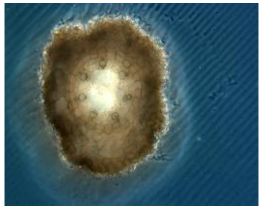	Quncho_42_ top_150um_10x_1 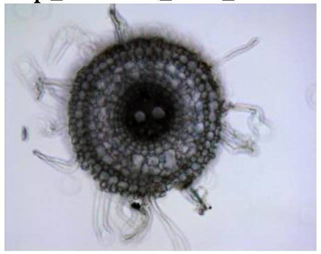	Quncho_20_ top_200um_20x_2 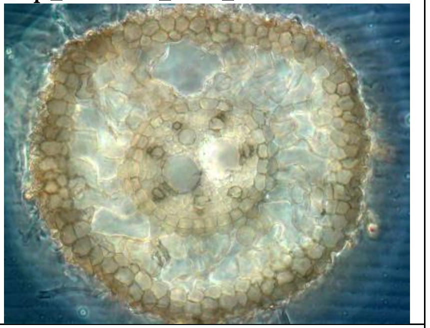
24	Q34_unten_2_10 x 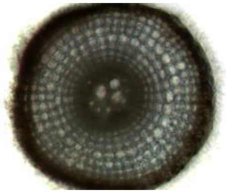	Q13_unten_1_10x 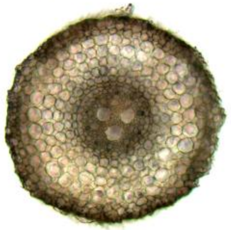	Q34_mitte_3_10x 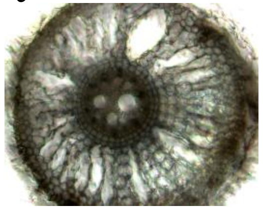	Q13_mitte_1_10x 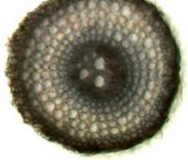	Q34_oben_1_10x 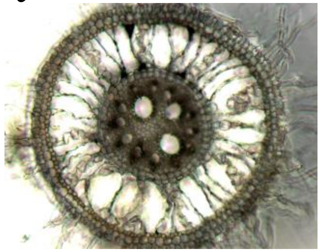	Q13_oben_1_10x 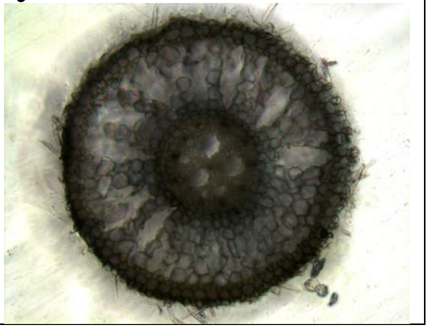

Days after treat ment	tip				top	
	control	waterlog	control	waterlog	control	waterlog
2<br>20x	Tsedey_58_tip_1502_150um_20x<br>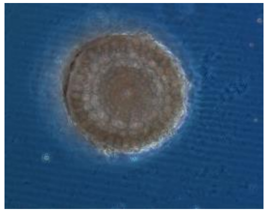	Tsedey_8_tip_1502_150um_20x<br>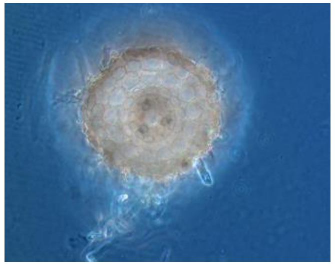			Tsedey_58_up_1502_200um_20x<br>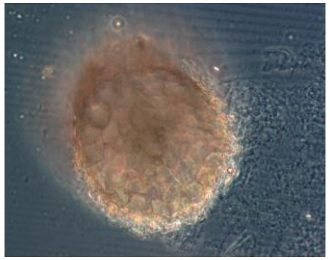	Tsedey_8_up_1502_150um_20x<br>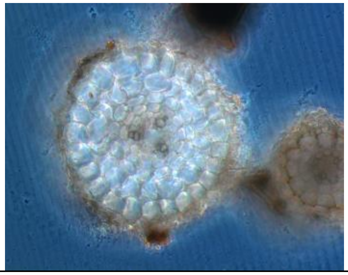
4<br>20x	Tsedey_66_tip_1702_150um_20x<br>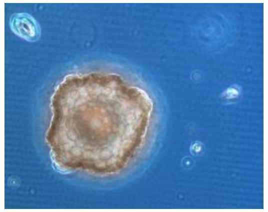	Tsedey_29_2nd root middle_1702_150um_20x.tif<br>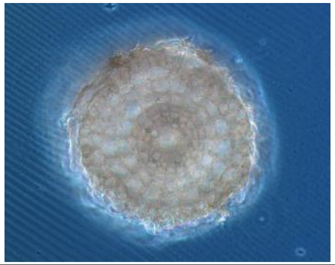			Tsedey_66_up_1702_150um_20x<br>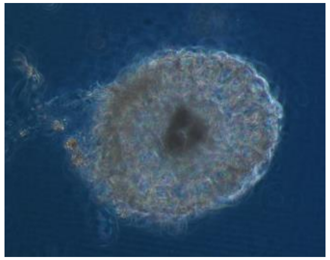	Tsedey_29_1st root up_1702_150um_20x.tif<br>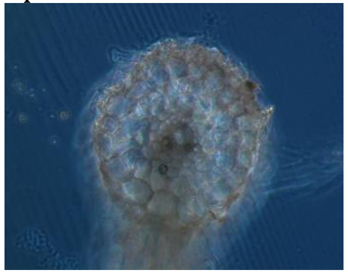
7	Tsedey_49_2nd tip_150_20x<br>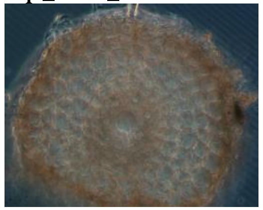		Tsedey_49_2nd middle_150_20x<br>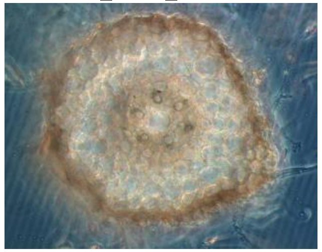	Tsedey_32_2nd middle_no_20x<br>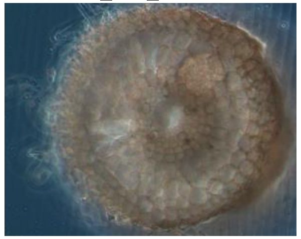	Tsedey_49_2nd up_150_20x<br>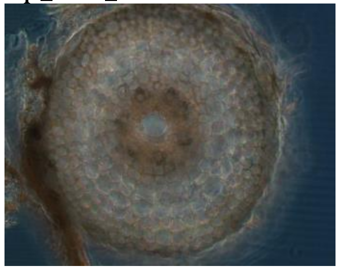	Tsedey_32_2nd up_150_20x<br>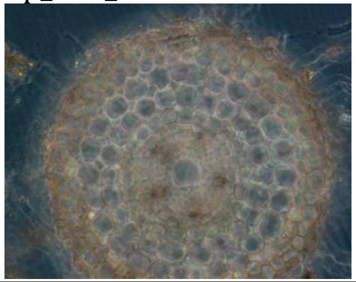
9<br>20x	Tsedey_45_2nd tip_150um_20x_1	Tsedey_19_2nd tip_150um_20x_2		Tsedey_19_2nd middle_150um_20x_2	Tsedey_45_2nd top_200um_20x_2	Tsedey_19_2nd top_200um_20x_1

	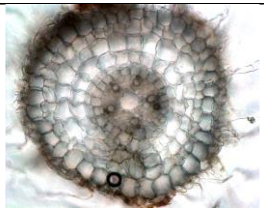	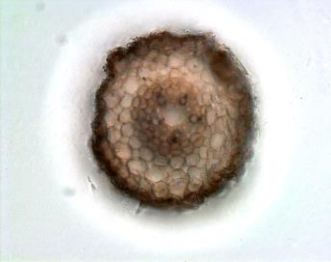		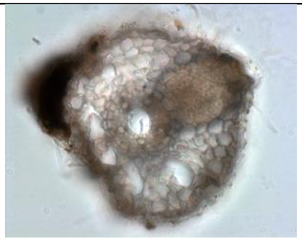	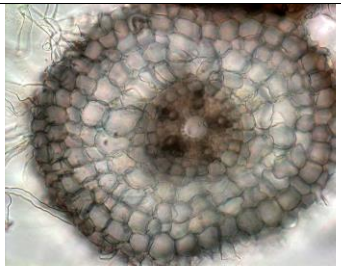	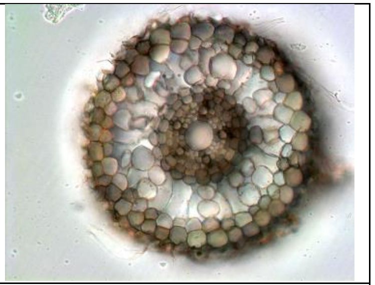
16	Tsedey_42_ tip_150um_10x_1 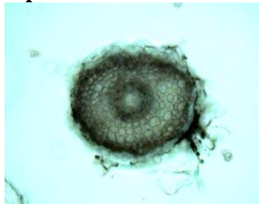	Tsedey_20_ tip_250um_10x 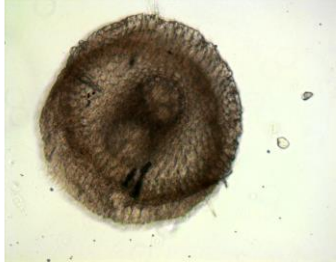	Tsedey_42_ middle_150um_10x_1 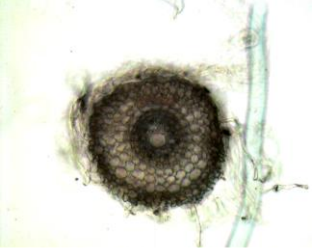	Tsedey_20_ middle_300um_10x_ 2 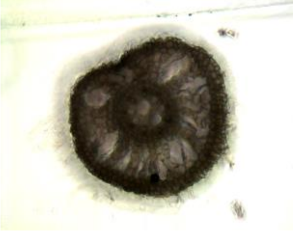	Tsedey_42_ top_150um_10x_1a.tif 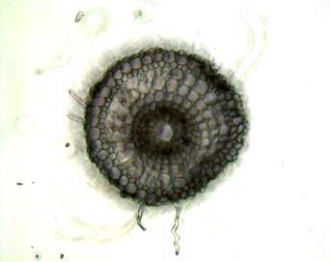	Tsedey_20_ top_250um_10x_2 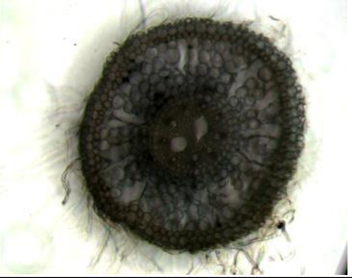
24	T34_unten_1_10x 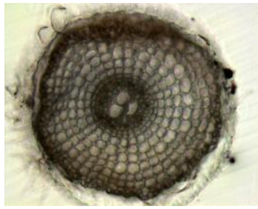	T13_unten_1_10x 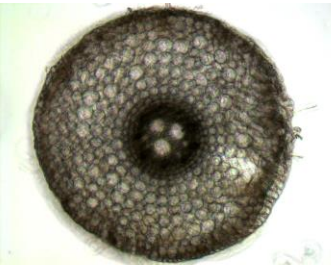	T34_mitte_5_10x 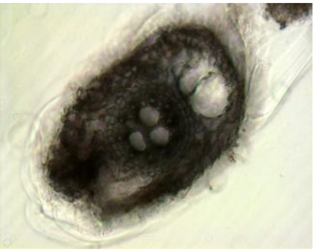	T13_mitte_1_10x 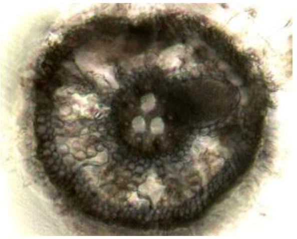	T34_oben_2_10x 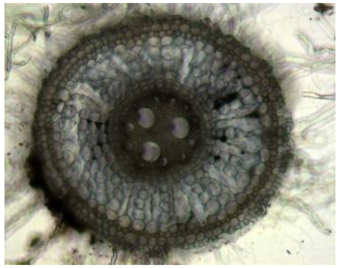	T13_oben_2_10x 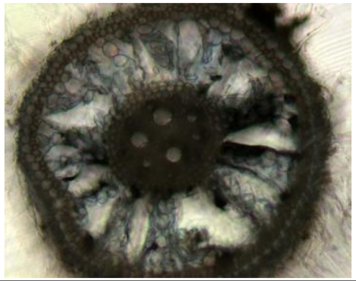

Figure S6. Complete set of cross sections taken at various time points after waterlogging. Cross sections of 4 day-old *Tsedey* and *Quncho* after 2, 4, 7, 9, 16 and 24 days after waterlogging.

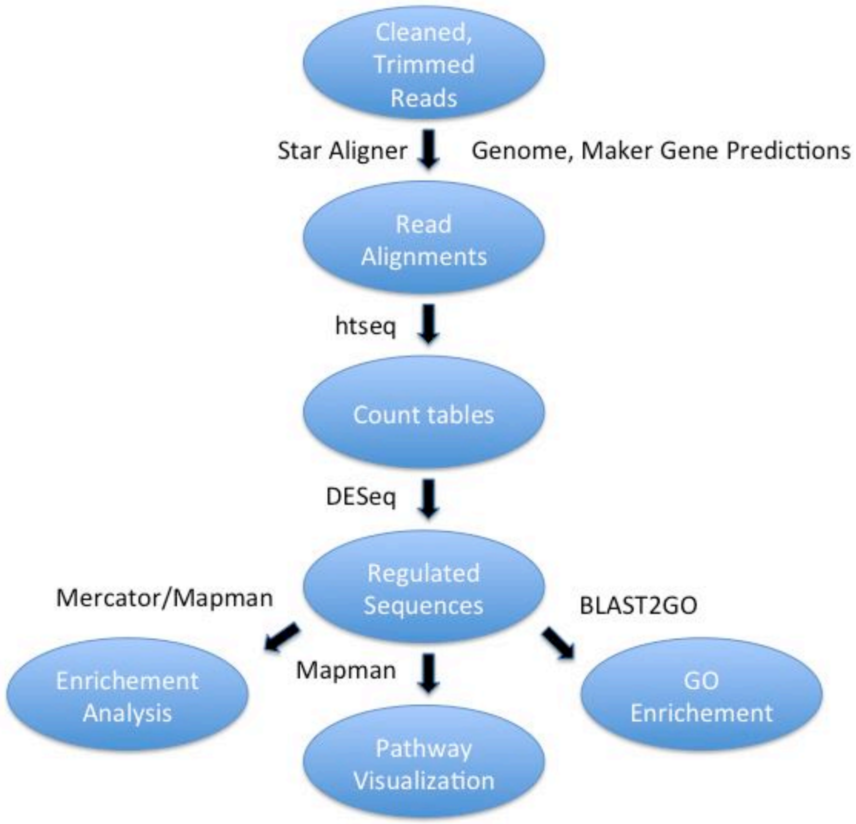

Figure S7. Pipeline for RNA-Seq Analysis. Raw reads were obtained for 2 conditions, normal watering (control) and waterlogging. The reads were cleaned, trimmed and mapped with the Star Aligner onto the tef genome. Count tables were generated with htseq, which was supplied with the genome, the predicted gene locations and the read alignments. The count tables were used as input to DESeq to find the differentially regulated genes. Pathways and functional categories enriched in regulated sequences were found using MapMan. Enriched GO categories were discovered using Blast2GO.

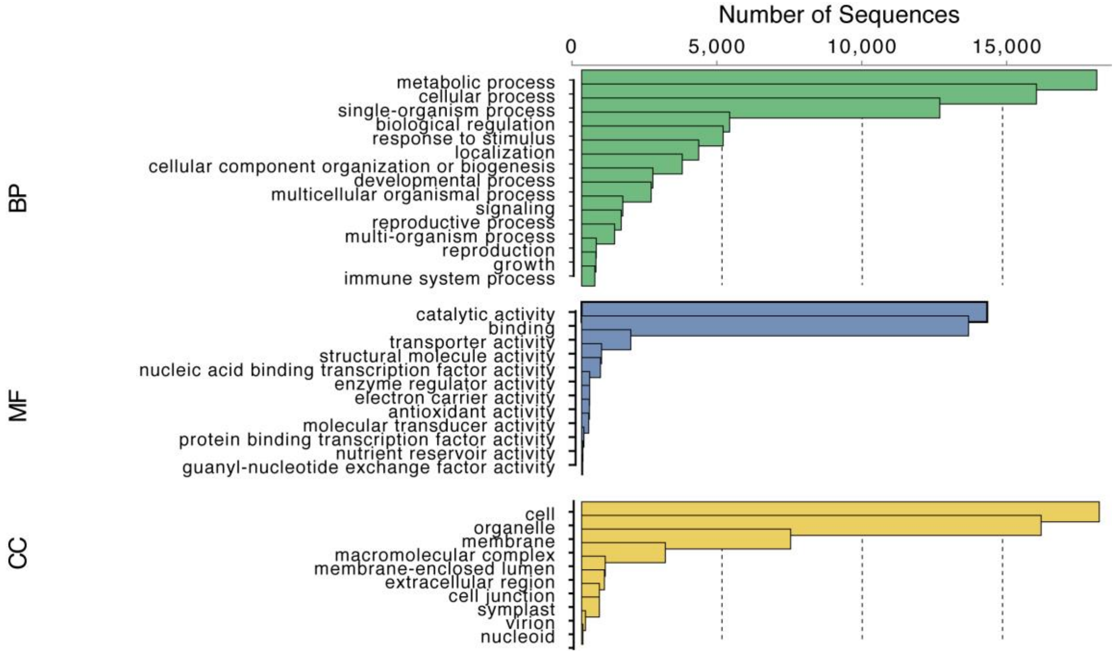

Figure S8. Distribution of GO Ontologies in the background set. The top functional categories of the GO Ontologies are shown for all proteins in the background set, included are the biological process (BP), molecular function (MF) and cellular component (CC).

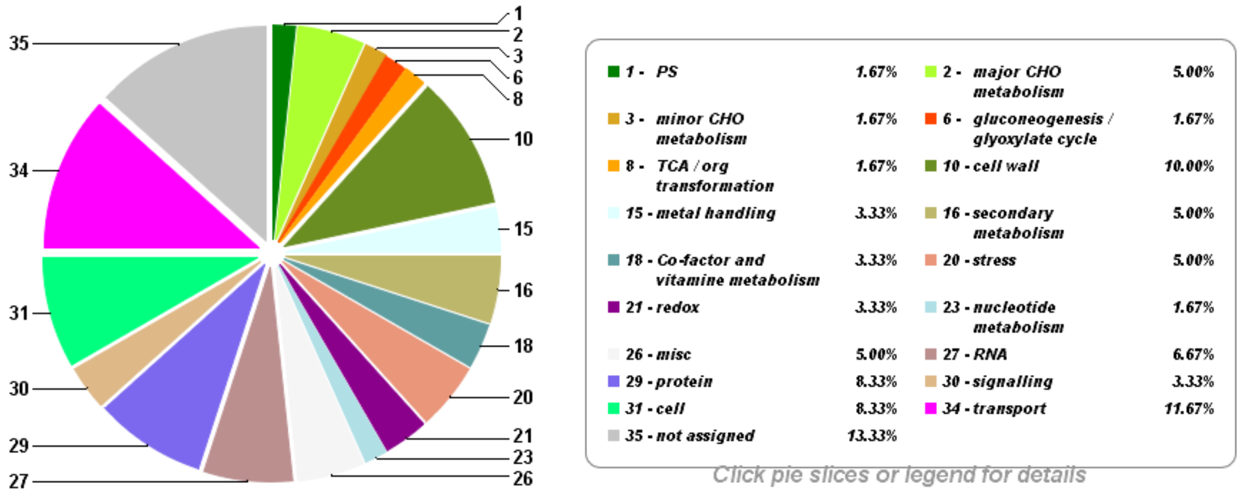

Figure S9. Distribution of protein functional categories in the background set as determine by Mercator. The Functional categories of transport (11.67%), cell wall (10.00%), CHO metabolism, major and minor (6.67%) and various other metabolisms are highly represented in the background set. PS- photosynthesis, redox- oxidation reduction, OPP- oxidative pentose phosphate, minor CHO metabolism- minor carbohydrate metabolism, misc- miscellaneous. Numbers represent the Mercator protein category while the percentages represent the percentage of total proteins in each category.

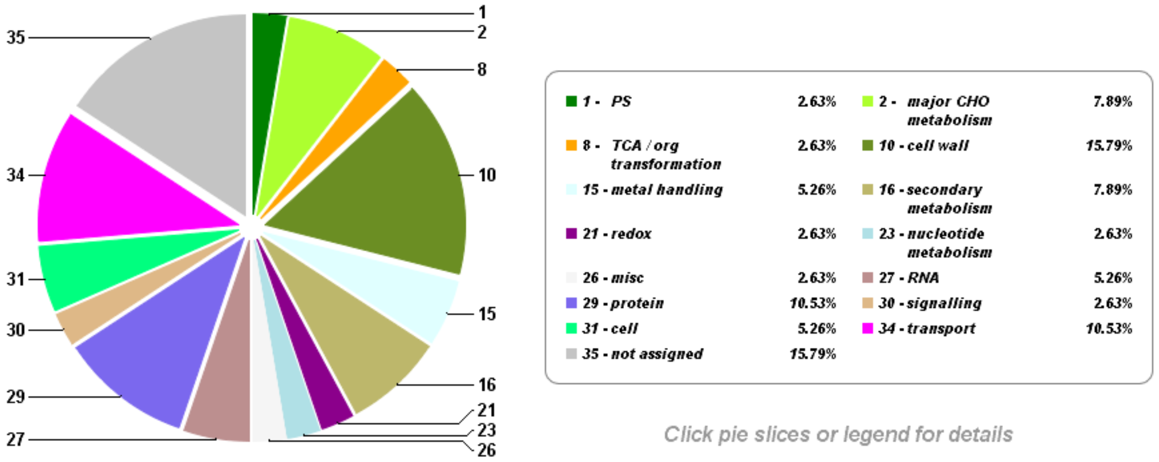

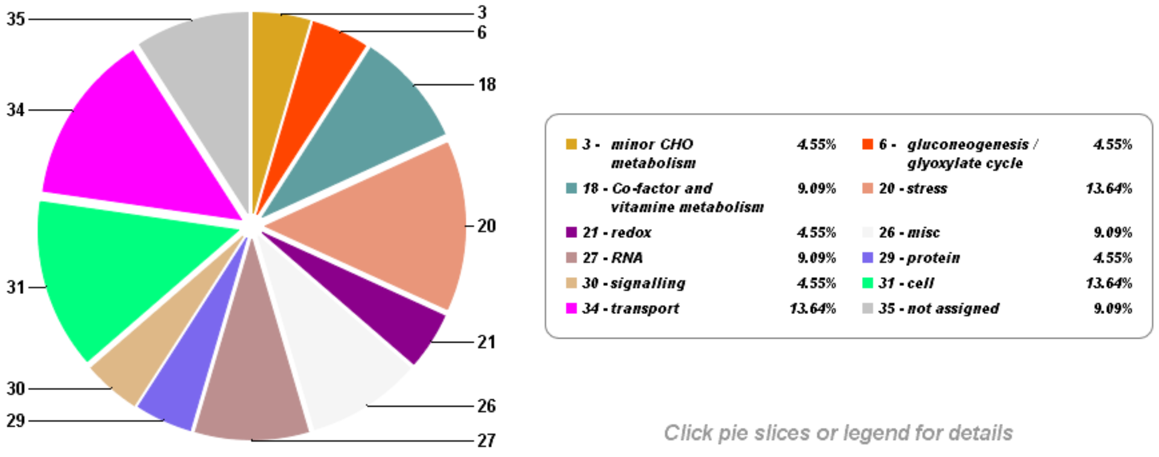

Figure S10. Differentially regulated tef proteins under waterlogging. Distribution of functional categories of (A) upregulated and (B) downregulated proteins as determined by Mercator. PS- photosynthesis, redox- oxidation reduction, OPP- oxidative pentose phosphate, minor CHO metabolism- minor carbohydrate metabolism, misc- miscellaneous. The Mercator protein category and the percentage of total proteins in that category are reported.

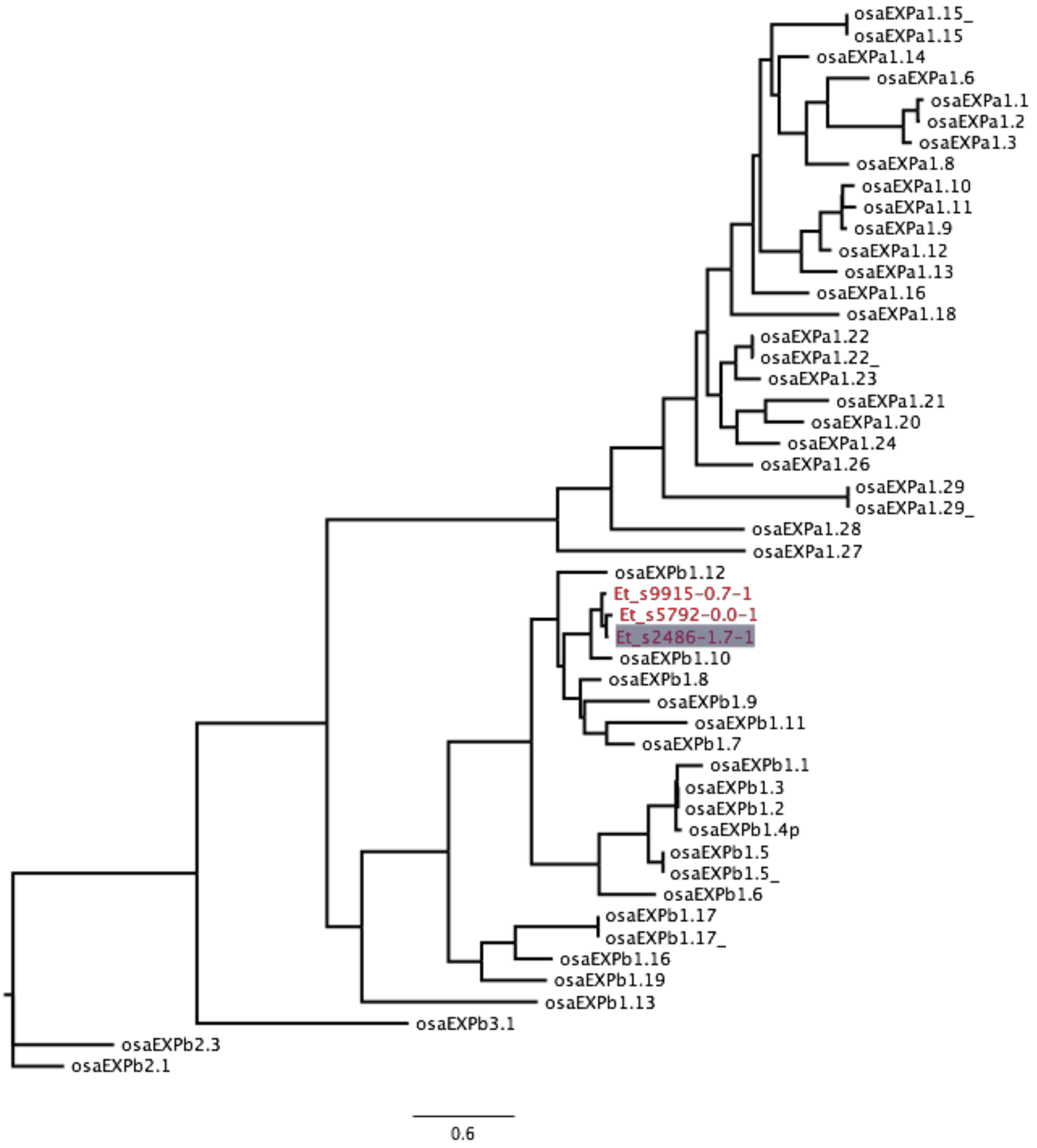

Figure S11. Phylogenetic tree of expansins. The tef expansin sequences (red) are upregulated under waterlogging and are of the type β1. They are most closely related to the rice sequence EXPβ1.10 and form a clade with EXPβ1.7, EXPβ1.8, EXPβ1.9 and EXPβ1.11 using the nomenclature described in the text.

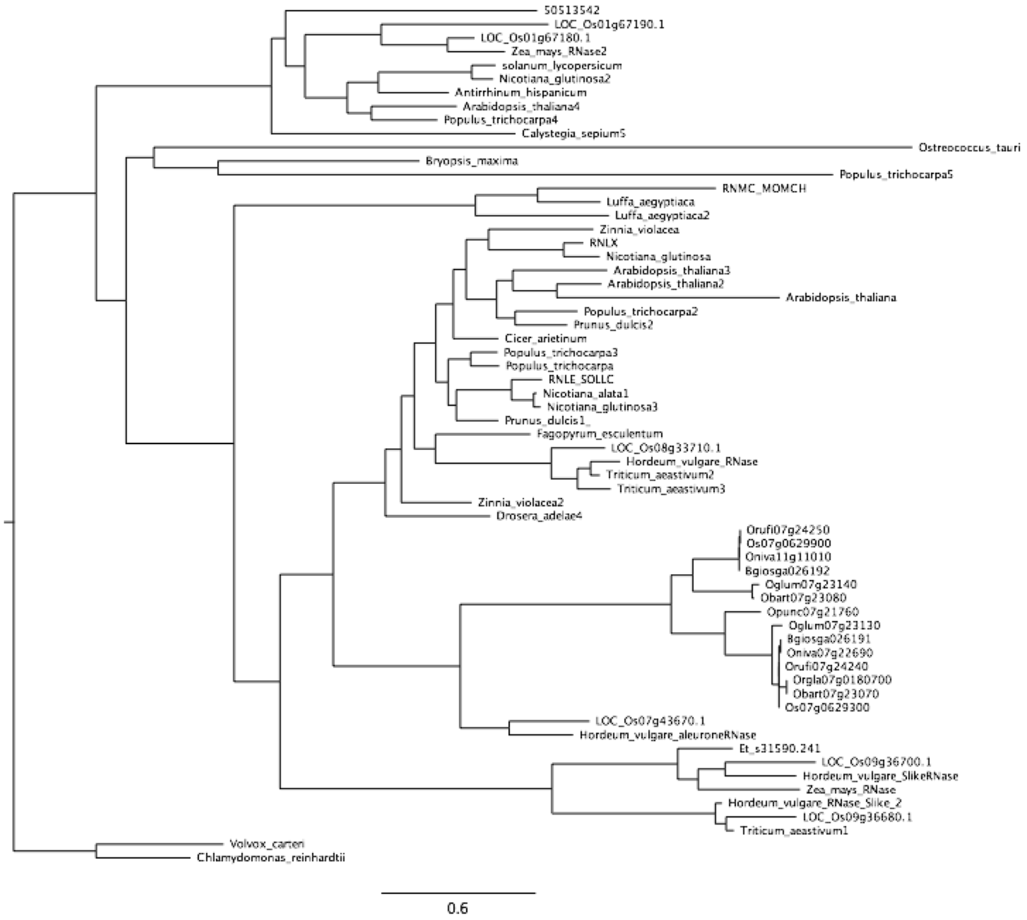

Figure S12. Phylogenetic tree of ribonuclease. The tef ribonuclease sequence Et_s3159-0.24 is downregulated under waterlogging stress and clusters with the monocot-specific Class I S-like clade. This clade includes proteins that have lost their ribonuclease activity but are expressed.

Supplementary Tables

Table S1. All data concerning shoot length, shoot dry weight, root length, root dry length, and number of leaves of *Quncho* subjected to early waterlogging. (**Excel file**)

Table S2. All data concerning shoot length, shoot dry weight, root length, root dry length, and number of leaves of *Tsedey* subjected to early waterlogging. (**Excel file**)

Table S3. Number of Adventitious Roots of *Alba*, *Quncho* and *Tsedey* after 9 days of waterlogging at the tillering stage.

Parameter	*Alba*			*Quncho*			*Tsedey*		
	Control	Waterlog	% of control	Control	Waterlog	% of control	Control	Waterlog	% of control
Number of adventitious roots (observation 1)	3.2 ± 1.1	3.5 ± 0.8	**109**	2.9 ± 1.1	3.7 ± 0.8	**128**	3.4 ± 0.5	3.9 ±1.0	**114**
Number of adventitious roots (observation 2)	2.8 ± 0.8	4.5 ± 0.8	**160***	2.9 ± 0.7	4.3 ± 1.0	**148***	1.9 ± 1.2	6.4 ± 1.0	**336***

Nineteen day-old plants were grown in soil with either normal watering or with water maintained at 1 cm below the soil surface. All values for numbers of roots are means (n=10 or 11) ± SD. Means marked with an asterisk are significantly different from each other (Mann-Whitney: *p<=0.05).

Table S4. Blast2GO annotations of predicted genes in the genome of *Eragrostis tef* (included as a separate text file). This is the full set of annotations of the genes predicted by Maker by the Tef Improvement Project. (**Excel file**)

Table S5. Genes differentially regulated when subjected to waterlogging stress, their counts and annotations. (**Excel file**)

Table S6. Cq values from the qPCR validation of the RNASeq expression values. (**Excel file**)
