# Supplementary material for: Waterlogging affects plant morphology and the expression of key genes in tef (Eragrostis tef)
Source: Plant Direct. 2018 Apr 25;2(4):e00056. doi: 10.1002/pld3.56 (PMC6508588; doi:10.1002/pld3.56)
Supplement: Supplementary file 4 [file PLD3-2-e00056-s004.docx]

**Table S3.** Number of Adventitious Roots of *Alba*, *Quncho* and *Tsedey* after 9 days of waterlogging at the tillering stage.

| Parameter | Alba | | | Quncho | | | Tsedey | | |
| --- | --- | --- | --- | --- | --- | --- | --- | --- | --- |
|  | Control | Waterlog | % of control | Control | Waterlog | % of control | Control | Waterlog | % of control |
| Number of adventitious roots (observation 1) | 3.2 ± 1.1 | 3.5 ± 0.8 | **109** | 2.9 ± 1.1 | 3.7 ± 0.8 | **128** | 3.4 ± 0.5 | 3.9 ±1.0 | **114** |
| Number of adventitious roots (observation 2) | 2.8 ± 0.8 | 4.5 ± 0.8 | **160*** | 2.9 ± 0.7 | 4.3 ± 1.0 | **148*** | 1.9 ± 1.2 | 6.4 ± 1.0 | **336*** |

Nineteen day-old plants were grown in soil with either normal watering or with water maintained at 1 cm below the soil surface. All values for numbers of roots are means (n=10 or 11) ± SD. Values marked with an asterisk are significantly different from each other (Mann- Whitney: *p<=0.05).
