# Supplementary material for: Waterlogging affects plant morphology and the expression of key genes in tef (Eragrostis tef)
Source: Plant Direct. 2018 Apr 25;2(4):e00056. doi: 10.1002/pld3.56 (PMC6508588; doi:10.1002/pld3.56)
Supplement: Supplementary file 8 [file PLD3-2-e00056-s008.pdf]

Authors response to reviewers and editors

(Plant Direct)

Reviewer #2: The manuscript has been improved through the changes made to the earlier version. There are still a few minor things that need attention

Reviewer comments	Authors response
Figure 1 it is a little hard to see the difference between the control and waterlogged - maybe put these in different colours rather than black and grey so they stand out a little more - at least for the on-line version if not the print copy?	Figure 1 is now available in colour
Pg10 and elsewhere should htseq be HTSeq throughout?	corrected
P25-26, table 5 - tubby is usually in capitals ie., TUBBY, TUBBY-like	done
Pg 26 KN1 should be spelt out as Knotted 1, Naphthylacetic acid not Napothylacetic acid	done
Pg 33 In the footnote 6 should be a superscript at the bottom	done
Conesa & Gotz (2008) on pg 17 is missing from the references	Included in the reference list
Pg 35 Zea nicaraguensis in italics	done
Pg 36 in Fukao et al -- ...submergence tolerance in rice.. delete the '(W)'; Hsu et al 2011 - - page number (e28888) missing?	corrected
Pg37 Juntawong et al 2014 - page number (658) missing; Kadam et al - page number (862) missing; Kawahara et al - page number (4) missing; Kayum et al - page number (23) missing; Lee et al 2011 Arabidopsis thaliana in italics	done
Pg 38 malik et al Hordeum marinum and triticum aestivum in italics; Miro et al Oryza sativa in italics; Reddy et al - no page number;	done
Pg 39 Sampedro & Cosgrove - no page numbers; Tatusov et al - no page numbers; Thirunavukkarasu et al - no page numbers; Tsuchiya et al - no page numbers; Watanabe et al - no page numbers;	corrected

Editor comments

In addition to the minor edits indicated by the reviewer, please check the reference section for the italicization of the species names suggested by the reviewer previously.	done
When changing "HTSeq Count" (Instead of htseq) please note it also needs to be changed in the footnotes of the tables, in addition to the locations suggested by the reviewer.	corrected
